# Di-[trioctyl-(8-phenyloctyl)-phosphonium] pamoate: synthesis and characterization of a novel, highly hydrophobic ionic liquid for the extraction of scandium, thorium and uranium

**DOI:** 10.3389/fchem.2024.1502232

**Published:** 2024-11-19

**Authors:** Andreas Gradwohl, Jakob Windisch, Alexander Rosner, Julia Heninger, Philipp L. Fuhrmann, Gabriele Wallner, Bernhard K. Keppler, Wolfgang Kandioller, Franz Jirsa

**Affiliations:** ^1^ Department of Inorganic Chemistry, University of Vienna, Vienna, Austria; ^2^ Department of Food Science and Technology, University of Natural Resources and Life Sciences, Vienna, Austria; ^3^ Department of Zoology, University of Johannesburg, Johannesburg, South Africa

**Keywords:** ionic liquid, task-specific, selective extraction, REE separation, leaching, green solvents

## Abstract

We synthesized and characterized a novel, task-specific ionic liquid for metal extraction with considerably reduced leaching behavior compared to similar, phosphonium-based ionic liquids. The synthesis involves the design of the novel compound [TOPP]_2_[PAM] featuring both a highly hydrophobic cation and a functional anion. The characterization of the novel ionic liquid confirmed the formation of the desired structure and sufficient purity. The high viscosity of [TOPP]_2_[PAM] is responsible for the comparably high working temperature of 50°C. Extraction experiments demonstrated the suitability of [TOPP]_2_[PAM] for extracting Sc, Th and U from aqueous matrices, whereby extraction efficacies of 87.3% ± 9.1% (Sc), 95.8% ± 2.3% (Th) and 92.7% ± 0.3% (U) were achieved over 24 h. Furthermore, Sc could be separated to a high degree via selective extraction from Th as well as from the rare earth elements Y, La, Ce, Nd, Eu, Ho and Lu. Th was separated from La, Ce, Nd, Eu, Ho and Lu at pH 1.00. During all extraction experiments, leaching into the aqueous extraction matrix peaked at only 0.134% ± 0.011% after 24 h. The loading capacities for [TOPP]_2_[PAM] differed between the investigated metals, the highest values being achieved for U. After extraction, 82.7% ± 2.8% of the extracted Sc could be recovered from the IL using nitric acid (10%), but less of Th and U.

## 1 Introduction

Metals are exploited by humans for numerous purposes and in extremely varying quantities. Their abundance in the Earth’s crust, however, differs greatly, influencing both availability and price. The demand for metals is expected to increase steadily, especially due to the emergence of so-called “green technologies,” which aim to reduce the dependence on carbon-based energy systems. The term critical metals has been introduced in this context to describe metals that may become scarce for routine use in the medium term. This scarcity can be understood as matter of degree rather than a particular metal being either critical or non-critical. Factors influencing the criticality can be geological or economic, but can also reflect technological evolution or environmental impacts. The group of critical metals include, *e.g.*, Co, Sb, In and the Rare Earth Elements (REE) (Graedel et al., 2014).

Scandium (Sc) is an element within the REE group and shares that group’s physico-chemical properties (Connelly et al., 2005; Balaram, 2019). Economically, it is considered the most expensive one (Junior et al., 2021). Junior et al. (2021) cite a price of 3800 US $ per kg scandium oxide (Sc_2_O_3_), whereby the second most expensive one is dysprosium oxide (Dy_2_O_3_) with a price of 180 US $ per kg. Interestingly, those authors report a recycling rate of zero for Sc, but between 3 and 8% for the other REE. In general, REE recycling and purification is considered to be expensive, inefficient and subject to certain limitations such as the uneven distribution of small amounts of elements within a much bigger matrix. This translates into a large, untapped potential in the development of strategies for economically sensible and environmentally neutral or beneficial recycling technologies of REE (Balaram, 2019). The purification of REE is often mentioned together with a separation of these elements from radioactive thorium (Th) and uranium (U). Bastnäsite and monazite contain up to 20% ThO_2_ and up to 16% UO_2_. Thorium is a major concern during REE production due to its radioactivity (Zhu et al., 2015). The main primary resources for Sc are thortveitite and lolbeckite ores, which are Sc silicate-rich ores that contain up to 45% Sc but are comparatively rare. Scandium is extracted from those ores via fractionation sublimation using chloride to obtain scandium chloride (ScCl_3_) at 967°C – a process considered neither sustainable nor ecofriendly. As those Sc-containing ores are so scarce, five times more Sc is produced from secondary resources, *e.g.*, from ores from the Bayan Obo mine in China, which contain, besides Sc, mainly other REE. It is also obtained from residues, where it represents a co-product (Junior et al., 2021). This scarcity, the detrimental environmental impact of traditional mining techniques and the increasing demand motivate the research interest in a greener way to exploit and purify REE. A promising, potentially greener way for production, purification and recycling of metals in general, including Sc, Th and U, is based on using ionic liquids as selective and reusable extraction agents from aqueous matrices (Stojanovic and Keppler, 2012; Janssen et al., 2015; Wang et al., 2017; Hidayah and Abidin, 2018; Junior et al., 2021; Yudaev and Chistyakov, 2022; Jirsa and López-López, 2023).

Ionic liquids (ILs) are commonly described as low-melting salts and consist of large, non-symmetrical organic cations and organic or inorganic anions. The definition often includes a melting point <100°C. ILs that are liquid at room temperature are referred to as room-temperature ILs (RTILs). In general, ILs display unique properties such as high chemical and thermal stability, low inflammability and especially a low vapor pressure. Those properties explain why ILs are often considered “greener” solvents. It is assumed that about 10^18^ combinations of ILs are possible, opening the door to directly designing and customizing the unique properties of ILs, *e.g.*, tuning them for metal extraction. ILs designed for a specific task are referred to as task-specific ILs (TSILs) (Seddon, 1997; Rogers and Seddon, 2003; Stojanovic and Keppler, 2012; Janssen et al., 2015; Welton, 2018; Jirsa and López-López, 2023).

Extraction of metals using ILs has been explored extensively in the literature, focusing on a high extraction efficacy and selectivity towards specific metals as well as on the applicability in varying aqueous matrices (Stojanovic and Keppler, 2012; Yudaev and Chistyakov, 2022; Jirsa and López-López, 2023). In most cases, the extraction mechanism is not fully understood and apparently relies on neutral extraction, ion exchange or a combination of both (Janssen et al., 2015). Recent examples for using TSILs for REE, Th and U extraction include the works of Scheiflinger et al. (2019), Sert and Yusan (2023), Gradwohl et al. (2023), Su et al. (2024) and Kaim-Sevalneva et al. (2024). Scheiflinger et al. (2019) used 10 different TSILs based on the common IL cations trihexyltetradecylphosphonium, methyltrioctylammonium and methyltrioctylphosphonium and the anions anthranilate, 2-hydroxy-5-nitrobenzoate, 2-(hexylthio)acetate, 4-aminosalicylate and thiosalicylate to extract the radionuclides Th, U, Po and Ra under varying pH conditions. They achieved extraction efficacies approaching 100% for U for all of the used ILs. Sert and Yusan (2023) successfully extracted Th from acidic aqueous solutions using the commercially available IL Cyphos^®^ IL 101. The authors were additionally able to separate Th from La(III) and Ce(III) respectively. Gradwohl et al. (2023) demonstrated the successful extraction of selected REE using the TSIL trihexyltetradecylphosphonium 3-hydroxy-2-naphthoate. Su et al. (2024) used two novel betaine derivatives to extract Sc, obtaining high yields. Kaim-Sevalneva et al. (2024) used quaternary ammonium-based ILs and achieved a separation of various lanthanides from Sc. All aforementioned authors highlighted the efficient applicability in metal extraction.

Far less attention has been paid to the leaching of ILs into the aquatic phase during the extraction process. Leaching limits their reusability and poses a potential risk of toxic effects when released into the environment (Platzer et al., 2017b; Flieger and Flieger, 2020; Pirkwieser et al., 2020; Jirsa and López-López, 2023). Although Pirkwieser et al. (2018) were able to reduce the leaching significantly compared to ILs with similar structures, a toxic effect towards algae remained (Pirkwieser et al., 2018; Pirkwieser et al., 2020). IL toxicity generally increases with their hydrophobicity, whereas an increased hydrophobic character of a comparably less toxic compound can help reduce toxicity (Ventura et al., 2013). Designing novel TSILs to contribute to an effective and greener way of recycling critical metals is therefore a balancing act between the potential of the unique properties of ILs and concerns regarding their (eco) toxicity.

Based on former works, we aimed to synthesize a novel TSIL with excellent extraction properties towards possibly toxic metals, yet showing minimized leaching behavior. We intended to synthesize a highly viscous compound that can be used undiluted. Furthermore, the compound should still feature the traditional scaffold of a quaternary phosphonium compound as cation, such as the Cyphos^®^ ILs (Bradaric et al., 2003), to remain comparable to the majority of ILs that have been established for metal extraction (Jirsa and López-López, 2023). In search of a more hydrophobic, non-toxic anion, we proposed pamoic acid as a promising precursor for a highly hydrophobic TSIL because it is practically insoluble in water (Stahl and Wermuth, 2002) but holds the salicylate functional group, which has proven to be very suitable for metal extraction (Fischer et al., 2011; Pirkwieser et al., 2018; Gradwohl et al., 2023).

In the present work, we describe the synthesis and characterization of the novel, task-specific ionic liquid Di-[trioctyl-(8-phenyloctyl)-phosphonium] pamoate, [TOPP]_2_[PAM], consisting of a novel, phosphonium-based cation with increased hydrophobicity and the anion pamoate. We therefore selected a tertiary phosphine with three octyl side chains and combined it with 1-chloro-8-phenyloctane. This enabled introducing a forth side chain that is even more hydrophobic than the other three, while at the same time providing the asymmetric character to the ionic liquid’s cation. The structure of the anion pamoate was selected as a consequent further development of the structural motif of 3-hydroxy-2-naphthoic acid (Pirkwieser et al., 2018; Gradwohl et al., 2023), from which it is synthetically derived by reaction with formaldehyde (Baghel and Rao, 2009). Literature reports on the acceptable pharmacological effects of pamoic acid (Zhao et al., 2010), its low solubility and its use in pharmaceuticals (Stahl and Wermuth, 2002) supported the decision for this anion.

The performance of this novel IL in extracting the metals Sc, Th and U was assessed in extraction experiments, and we present optimal conditions for such extraction from acidic aqueous solutions. We also demonstrate the selective extraction of Sc and Th in the presence of selected REE. Finally, we investigated the IL’s leaching behavior in order to assess the potential ecotoxicological impact and the applicability of [TOPP]_2_[PAM] as a greener extraction agent for metal extraction and separation from simple aqueous matrices.

## 2 Materials and methods

### 2.1 Solvents and reagents

The chemicals for synthesis – thionyl chloride (≥99%), potassium hydroxide (≥85%) and pamoic acid (≥97%) – were purchased from Sigma-Aldrich (United States), trioctylphosphine (98%) was purchased from Apollo Scientific (United Kingdom) and 8-phenyloctan-1-ol (97%) from abcr chemicals (Germany). The solvents used during synthesis, namely, methanol (HPLC grade, 99.8%) and dichloromethane (analytical reagent grade, 99.8%), were purchased from VWR (United States). Celite^®^535 (AlfaAesar, United States) is a diatomaceous-earth-based filtration material. Decolorization experiments were performed using activated charcoal (untreated, granular, 20–60 mesh; Sigma-Aldrich, United States).

Element standard solutions of Sc, Y, La, Ce, Eu and Lu (1,000 mg L^−1^ ± 4) in 2%–5% (w/w) HNO_3_ for preparing the extraction solutions were purchased from SigmaAldrich (United States), Ho (999 μg mL^−1^ ± 5) in 5% (w/w) HNO_3_ from AlfaAesar (United States). Th and U stock solutions were prepared from thorium nitrate (Merck, Germany) and uranyl nitrate (Merck, Germany) by dissolving the respective salts in 0.05 M nitric acid and subsequently used to spike the respective aqueous extraction matrices to a concentration of 10 or 20 mg L^−1^. The liquid scintillation (LSC) cocktail AquaLight (Hidex, Finland) was used for sample preparation for LSC measurements of Th and U. A Pt standard solution (1,000 mg L^−1^ in 10% (w/w) HCl; VWR, United States) was used for sample preparations before TXRF measurements. The polyvinyl alcohol (PVA, 0.3 g L^−1^) solution for TXRF measurements was prepared from hydrolyzed polyvinyl alcohol (86%–89%) with low molecular weight (AlfaAesar, United States). For pH adjustment and dilutions, we used sodium hydroxide (50% (w/w) in H_2_O, extra pure; Acros Organics, Belgium) and nitric acid (>68%, PrimarPlus-trace analysis grade; Fisher Scientific, United States). Ultra-pure water of resistivity >18.2 MΩ cm was obtained from a Millipore MilliQ apparatus (Merck Millipore, United States). Aliquat^®^ 336 and trihexyltetradecylphosphonium chloride were purchased from Sigma-Aldrich (United States) for additional, comparative leaching experiments.

### 2.2 Apparatus

The Initiator Microwave Synthesizer (Biotage, Sweden) was used for the last step of synthesis. The ionic liquid was characterized by ^1^H and ^31^P NMR analysis, mass spectrometry and elemental analysis. The NMR spectra were recorded on an Avance III™ 500 MHz spectrometer (Bruker, United States) in DMSO at 298.1K using pulse programs at 500.10 MHz (^1^H) and 202.44 MHz (^31^P). Mass spectra were recorded on a timsTOF flex (Bruker, United States) in Acetonitrile/Methanol +1% H_2_O. Elemental Analysis was performed using an EA 3000 CHNS-O Elemental Analyzer (Eurovector, Italy) and an Agilent 7100 CE capillary electrophoresis instrument (Agilent, United States) with a TraceDec conductivity detector was used to determine the chloride content. Phosphorus analysis was done photometrically via formation of Molybdenum blue. The density of the ionic liquid was determined with a Gay-Lussac calibrated glass pycnometer (Blaubrand^®^, Brand, Germany). Shear and oscillatory rheological properties were characterised using an MCR 302 rheometer (Anton Paar, Austria) using a 50 mm cone-plate geometry with a 1° angle, at a gap of 0.104 mm. Th and U concentrations were determined with the liquid scintillation (LSC) counter Hidex 300 SL (Hidex, Finland). The REE metal content was determined using the total-reflection X-ray fluorescence (TXRF) spectrometer S2 PICOFOX (Bruker, United States) and quartz glass sample carrier discs. Total organic carbon (TOC) was determined using a TOC-L analyzer (Shimadzu, Japan). pH values were determined using a ProLab 2000 pH meter (Schott Instruments, Germany). All masses were weighed using a METTLER AT200 analytical balance (Mettler-Toledo, United States). Samples were shaken for extraction on a Vibramax 100 orbital shaker (Heidolph, Germany). Constant temperature during temperature-dependent extraction experiments was assured by placing the orbital shaker in either a 20°C thermostat cabinet (TS 606/2, WTW, United States) or a heating cabinet (TK/L 4250, Ehret, Germany).

### 2.3 Synthesis of the ionic liquid

#### 2.3.1 1-Chloro-8-phenyloctane

8-Phenyloctan-1-ol (10.0 g, 1 eq.) was transferred to a round-bottom flask and stirred at room temperature. Thionyl chloride (17.3 g, 3 eq.) was added, stirred and heated for 12 h at 50°C. The crude product was distilled *in vacuo* (*p* = 0.2 mbar, T = 150°C) and the product stored under Ar until further use. Yield: 94%, colorless liquid. ^1^H NMR (500.10 MHz, DMSO-*d*_6_): δ = 7.30–7.23 (m, 2H, H_arom_), 7.21–7.13 (m, 3H, H_arom_), 3.62 (t, *J* = 6.6 Hz, 2H, –CH_2_–Cl), 2.60–2.53 (m, 2H, C_arom_–CH2–), 1.74–1.66 (m, 2H, –CH_2_–), 1.61–1.50 (m, 2H, –CH_2_–), 1.40–1.22 (m, 8H, –CH_2_–).

#### 2.3.2 Trioctyl-(8-phenyloctyl)-phosphonium chloride, [TOPP]Cl

Trioctylphosphine (5.00 g, 1.0 eq.) was transferred to a round-bottom flask, set under Ar and stirred at room temperature. 1-Chloro-8-phenyloctane (4.56 g, 1.5 eq.) was added and the mixture was heated at 180°C for 96 h. After the reaction, the crude product was distilled *in vacuo* (*p* = 0.2 mbar, T = 180°C) for 6 h to remove excessive 1-chloro-8-phenyloctane. Yield: 98%, dark-orange viscous liquid. ^1^H NMR (500.10 MHz, DMSO-*d*_6_): δ = 7.30–7.23 (m, 2H, H_arom_), 7.17 (d, *J* = 7.5 Hz, 3H, H_arom_), 2.57 (t, *J* = 7.7 Hz, 2H, C_arom_–CH_2_–), 2.18 (ddd, *J* = 16.9, 10.2, 6.1 Hz, 8H, P–CH_2_–), 1.64–1.17 (m, 48H, –CH_2_–), 1.02–0.67 (m, 9H, –CH_3_). ^31^P NMR (202.44 MHz, DMSO-d_6_): δ = 33.83.

#### 2.3.3 Di-[trioctyl-(8-phenyloctyl)-phosphonium] pamoate, [TOPP]_2_[PAM]

Pamoic acid (1.142 g, 1.0 eq.) was transferred to a 20 mL microwave flask and dispersed in methanol. Potassium hydroxide (0.500 g, 3 eq.) was dissolved in methanol, added dropwise to the pamoic acid dispersion and the reaction mixture was stirred for 30 min. Then, trioctyl-(8-phenyloctyl)-phosphonium chloride (3.500 g, 2.0 eq.) was dissolved in methanol and added to the reaction mixture. The microwave flask was sealed and heated for 6 min to 100°C in the Initiator Microwave Synthesizer (Biotage, Sweden). Finally, methanol was removed under reduced pressure and the residue was dissolved in dichloromethane and filtered over Celite^®^535. The pure product di [trioctyl-(8-phenyloctyl)-phosphonium] pamoate, [TOPP]_2_[PAM], was concentrated under reduced pressure and dried *in vacuo* at 50°C for 72 h. Yield: 90%, dark brown highly viscous liquid. ^1^H NMR (500.10 MHz, DMSO-d_6_): δ = 8.21 (d, *J* = 8.6 Hz, 2H, H_arom_), 8.13 (s, 2H, H_arom_), 7.63–7.57 (m, 2H, H_arom_), 7.30–7.23 (m, 4H, H_arom_), 7.17 (d, *J* = 7.3 Hz, 6H, H_arom_), 7.08 (ddd, *J* = 8.4, 6.7, 1.4 Hz, 2H, H_arom_), 6.97 (dd, *J* = 7.3, 7.3 Hz, 2H, H_arom_), 4.66 (s, 2H, C_arom_–CH_2_–C_arom_), 2.56 (t, *J* = 7.7 Hz, 4H, C_arom_–CH_2_–), 2.16 (td, *J* = 13.1, 7.5 Hz, 16H, P–CH_2_–), 1.64–1.17 (m, 96H, –CH_2_–), 0.86 (td, *J* = 6.9, 2.7 Hz, 18H, –CH_3_). ^31^P NMR (202.44 MHz, DMSO-d_6_): δ = 33.8.

### 2.4 Extraction and back-extraction experiments

Aqueous extraction matrices were prepared by diluting the respective metal standard solutions with nitric acid (2%). Each aqueous phase contained 10 mg L^−1^ Sc or Th or 20 mg L^−1^ U, respectively, at the beginning of the experiment. The pH was set to 1.00 ± 0.03 or 3.00 ± 0.03 using sodium hydroxide solutions (0.5%, 5.0% and 50% (w/w)). For multi-element extraction experiments, two different lines of experiments were conducted. On the one hand, nitric acid (2%) was spiked with 10 mg L^−1^ Sc and 10 mg L^−1^ Y, La, Ce, Eu, Ho or Lu, on the other hand nitric acid (2%) was spiked with 10 mg L^−1^ Th and 10 mg L^−1^ Sc, La, Ce, Eu, Ho or Lu. The pH was brought to pH = 1.00 ± 0.03 (Th) or pH = 3.00 ± 0.03 (Sc) using sodium hydroxide dilutions. All extraction experiments were carried out in triplicates, including reference triplicates of extraction matrix without IL for determination of metal stability during the respective time.

The IL di-[trioctyl-(8-phenyloctyl)-phosphonium] pamoate was weighed into 50 mL polypropylene centrifuge tubes (100 ± 10 mg) using an analytical balance accurate to 0.1 mg. The vials were then centrifuged at 4,000 rpm at 40°C for 15 min to allow the IL to form a homogeneous drop at the bottom of the tube. 20 mL of prepared metal extraction matrix was added onto the IL and agitated for either 1 h, 2 h, 4 h, 6 h or 24 h using an orbital shaker (250 rpm). The experiments were conducted at either 20°C, 30°C, 40°C or 50°C, controlled by placing the orbital shaker either in a climate- (20°C) or heating cabinet (30°C, 40°C, 50°C). After the respective extraction time, an aliquot of 10 mL of the aqueous phase was removed with a syringe and filtered through a 0.45 µm PTFE syringe filter for further analysis.

**FIGURE 1:**
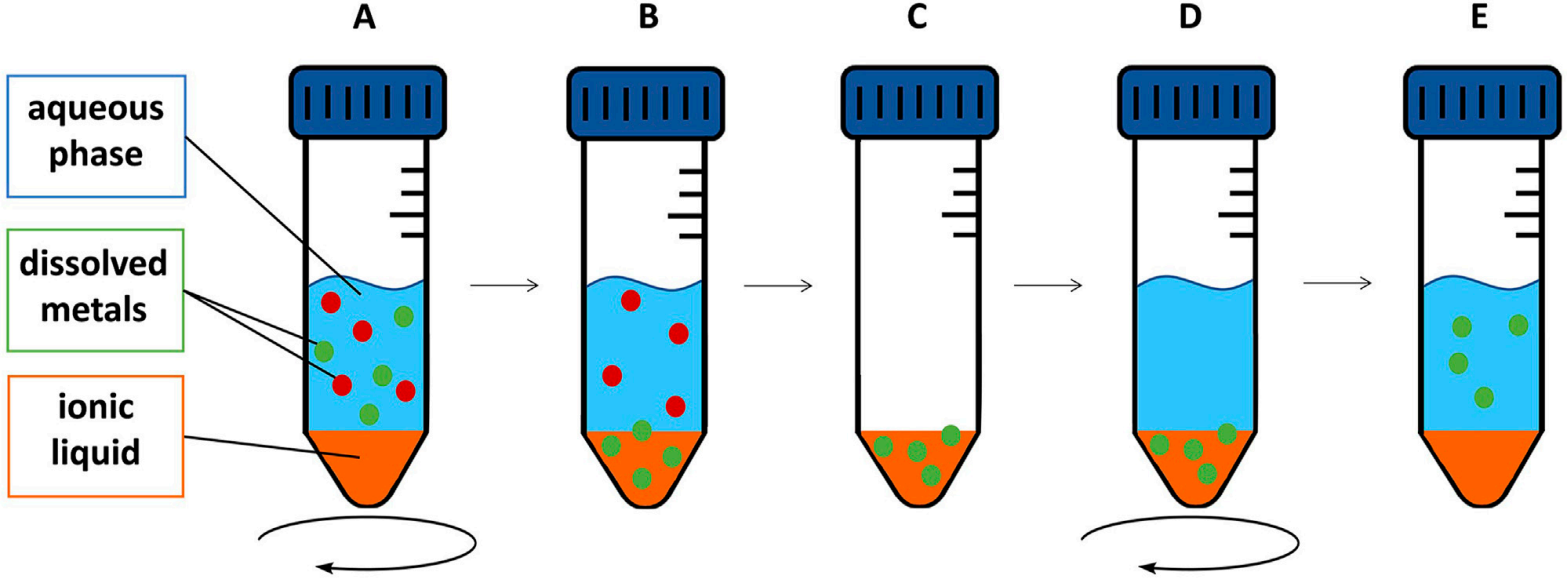
Schematic depiction of extraction and back-extraction experiments: IL and feed solution are agitated for a certain time **(A)**. After the respective time, metal is extracted into the IL selectively **(B)**. After removal of the aqueous phase **(C)**, a back-extraction agent was applied **(D)** to regain the metal after the respective back-extraction time **(E)**.

For back-extraction experiments, we used nitric acid dilutions (2%–10%) following Platzer et al. (2017a), who achieved best results using this agent, 0.5 M sulfuric acid or 0.03 M EDTA +0.5 M NaCl following Sert and Yusan (2023), as well as EDTA (1.0 M). All these experiments were performed in triplicates. After removing the extraction matrix of the extraction experiments, the metal-containing IL was used for the back-extraction experiments. The determined concentration in the extraction matrix was used to calculate the metal content in the IL. 20 mL back-extraction agent was pipetted onto the metal-containing IL and agitated for 24 h at 50°C using an orbital shaker (250 rpm). Then, an aliquot of 10 mL of the aqueous phase was removed with a syringe, filtered through a 0.45 µm PTFE syringe filter and stored for further analysis. The experimental setup, including extraction and back-extraction experiments, is shown in Figure 1.

### 2.5 Analysis and quantification

Viscosity ( η ) of the IL as a function of temperature was characterised by obtaining flow curves from 0.001 to 100 s^−1^, at desired temperatures from −5–70°C. The steady state viscosity at the Newtonian flow behaviour of the curves was extracted and plotted as a function of temperature, then fitted using the VFT Equation 1 (Tao and Simon, 2015). The constant A represents the asymptotic value of the viscosity (10^A^ mPa s), while B is a material-specific parameter. Amplitude and frequency sweeps were performed at 20°C and 70°C, at a frequency of 10 rad s^−1^ and an amplitude of 0.01%–100%, and a frequency of 100 to 0.1 rad s^−1^ and an amplitude of 0.5%, respectively.

$$\log10\left(\eta \right)=A+\frac{B}{T‐T_{0}}$$ (1)

The pH in the aqueous phase was determined in the filtered sample after the respective extraction. Th and U concentrations were determined using the Liquid Scintillation (LSC) Counter Hidex 300 SL counter (Hidex). Hereby, 4 mL of the filtered sample were transferred into 20 mL vials and mixed with 16 mL of the LSC cocktail AquaLight (Hidex). After 30 min cooling time at 5°C, the samples were measured for 4,000–12,000 s. TXRF measurements (S2 PICOFOX, Bruker) were performed to determine the REE metal content. Hereby, 0.5 mL of the filtered sample were mixed with 0.5 mL Pt standard solution (5 mg L^−1^ in 5% (w/w) HCl) and 100 µL of PVA solution (0.3 g/100 mL). A 5 µL aliquot of the sample was pipetted on a siliconized quartz glass sample carrier plate and dried for 40 min under an IR lamp under reduced pressure. After drying, TXRF spectra of the samples were recorded and evaluated using the software Spectra (Bruker, version 7.8.2.0). Recovery rates for REE using Pt as internal standard are published elsewhere (Gradwohl et al., 2023).

The extraction efficacy for metals from extraction matrices was calculated using Equation 2. It is defined as the percentage of removed metal from the extraction matrix after the respective extraction times in relation to the mean metal concentration in the reference samples. $\bar{c_{ref}}$ is the mean metal concentration of the reference samples, c_t_ the metal concentration in the respective sample after the extraction.

$$\text{Extraction efficacy }\left(\%\right)=\frac{\bar{c_{\text{ref}}}\;–{\;c}_{t}}{\bar{c_{\text{ref}}}}\cdot 100\;\left(\%\right)$$ (2)

In the case of double-element standards, where no secured decrease in metal concentration could be determined with the used analytical method, the confidence interval (95%, n = 6) of the measurements was determined with 5 degrees of freedom (n – 1) (Dunstan et al., 2018; Simundic, 2008). The mean ( $\bar{x},$ n = 6) and the lower limit of the confidence interval were used to calculate the highest possible extraction efficacy (≤%) following Equations 3, 4.

$$\text{Confidence Interval }\left(95\;\%\right)=\bar{x}\;\pm \;\left(2.015\cdot \frac{\sigma }{\sqrt{n}}\right)=\bar{x}\;\pm \;F$$ (3)

$$\text{Highest possible extraction efficacy }\left(\leq \%\right)=\frac{\bar{x}\;–\;\left(\bar{x}\;–\;F\right)}{\bar{x}}\cdot 100\;\left(\leq \%\right)$$ (4)

Leaching was determined based on total organic carbon (TOC) measurements. 5 mL of filtered sample were diluted with 5 mL MilliQ water and measured in a TOC-L analyser (Shimadzu). The measured TOC value was divided by the carbon content factor of the IL (C_IL_) to determine IL leaching using Equation 5. The carbon content factor equals 0.7894 and was derived from the carbon content of the used ionic liquid (78.94%). The leaching (%) was calculated using Equation 6, whereby V_s_. represents the volume (L) of the aqueous phase during extraction and m_IL_ the mass (mg) of used IL. The cation and anion of the IL were assumed to leach equally. Leaching therefore represents the percentual loss of IL during the extraction. For further comparison of the leaching of [TOPP]_2_[PAM] to commercially available, commonly used ILs, we investigated the leaching at 50°C of Aliquat^®^ 336, tricaprylmethylammonium chloride and trihexyltetradecylphosphonium chloride after a contact time of 24 h. These experiments were conducted in triplicates using 100 mg of the respective IL and 20 mL of pure water (MilliQ).

$$\text{Leaching }\left(\text{mg }L^{‐1}\right)=\frac{\text{TOC}}{C_{\text{IL}}}\;\left(\text{mg }L^{-1}\right)$$ (5)

$$\text{Leaching }\left(\%\right)=\frac{\text{TOC}\cdot V_{S}}{m_{\text{IL}}}\cdot 100\cdot \frac{1}{C_{\text{IL}}}\;\left(\%\right)$$ (6)

The back-extraction efficacy was calculated using Equation 7. It is defined as the percentage of metal in the aqueous phase after the back extraction in relation to the metal content in the used IL. The metal concentration after back extraction, c_aq_, is multiplied by the volume used for back extraction (V_be_). This equals the mass of REE in the aqueous phase, m_REE,aq_. The calculated value for the mass of REE is divided by the mass of REE in the IL (m_REE,org_) and multiplied by 100 to determine the efficacy of back-extraction. The REE mass in the organic phase (m_REE,org_) is calculated by using the measured concentration after the respective extraction, c_ext_, which is subtracted from the initial concentration of 10 mg L^−1^ and furthermore multiplied with the volume used during extraction, V_e_. The resulting value equals the mass of REE in the organic IL phase (m_REE,org_).

$$\text{Back}‐\text{extraction efficacy }\left(\%\right)=\frac{c_{\text{aq}}\cdot V_{\text{be}}}{\left(10\;–\;c_{\text{ext}}\right)\cdot V_{e}}\cdot 100=\frac{m_{\text{REE},\text{aq}}}{m_{\text{REE},\text{org}}}\cdot 100\;\left(\%\right)$$ (7)

Loading capacity was calculated following Scheiflinger et al. (2019) using Equation 8. In this equation, c_0_ refers to the initial metal concentration in the aqueous phase, c_t_ to the concentration after the extraction. The used volume of 20 mL (V) and the weighed-in mass of the IL (m) were then used to calculate the uptake in milligram metal per gram IL (Q_e_).

$$Q_{e}=\frac{\left(c_{0}\;–\;c_{t}\right)}{m}\cdot V$$ (8)

Distribution ratios (D) and separation factors were calculated in line with recent literature for enhanced comparability (Fu et al., 2021; Sert and Yusan, 2023; Kaim-Sevalneva et al., 2024). The distribution ratio between aqueous and organic (IL) phase was calculated using Equation 9. The separation factors for Sc or Th from the REE were then calculated from the corresponding distribution ratios using Equation 10. In the case of double-element standards, where no secured decrease in metal concentration could be determined with the used analytical method, the same approach as for the calculation of the highest possible extraction efficacy was used. The mean concentration (n = 6) and the lower limit of the confidence interval were used to calculate the lowest possible aqueous metal concentration and by that the highest possible metal concentration in the IL. This was the case for all double-element extractions of Th except the one with Sc and for the double-element experiments of Sc with Y, Ce or Lu.

$$D=\frac{c_{\text{IL}}}{c_{\text{aq}}}$$ (9)

$$\text{SF}=\frac{D_{\text{Sc},\text{Th}}}{D_{\text{REE}}}$$ (10)

## 3 Results and discussion

### 3.1 Synthesis and characterization of the ionic liquid [TOPP]_2_[PAM]

**FIGURE 2:**
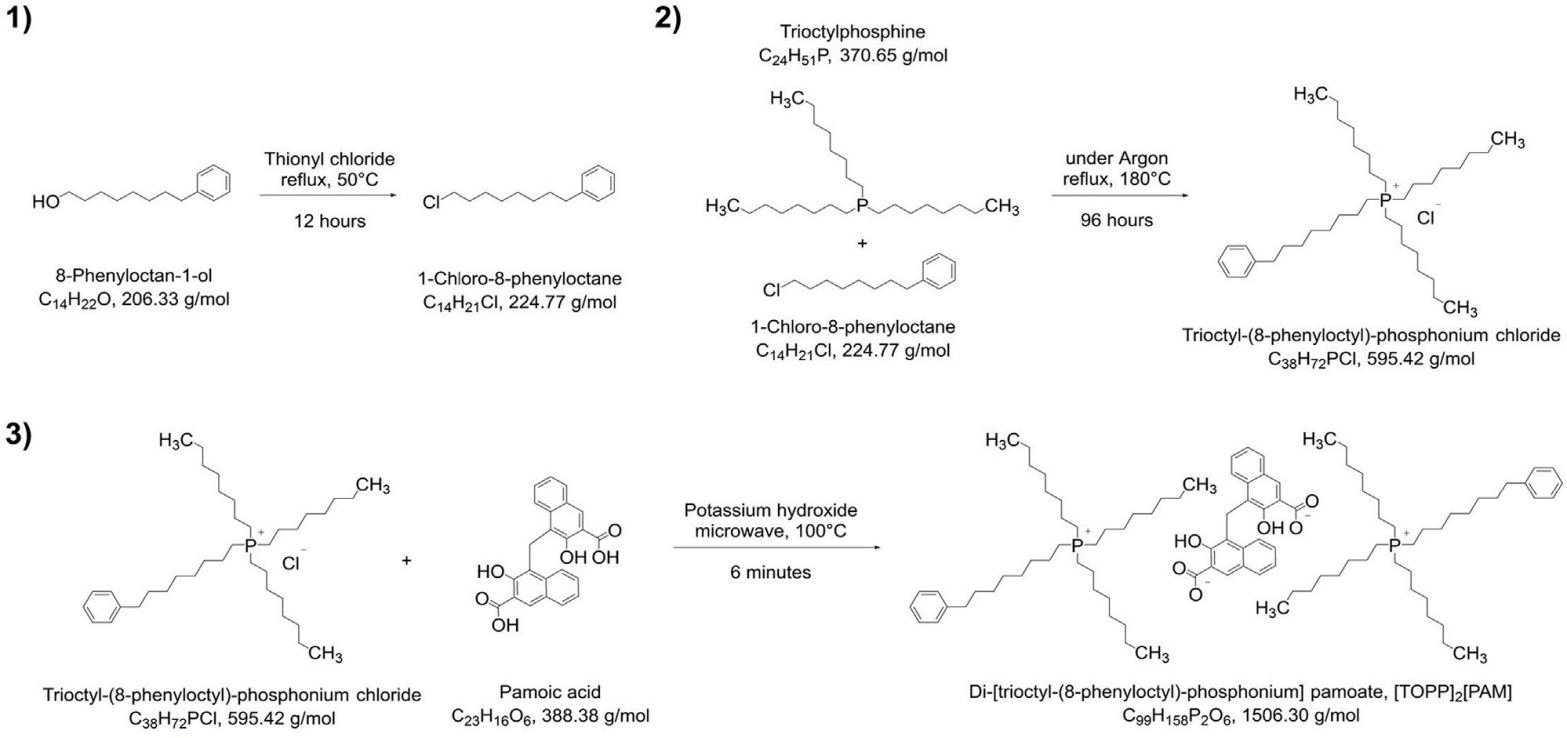
Synthesis pathway of [TOPP]_2_[PAM].

The synthesis of the IL di-[trioctyl-(8-phenyloctyl)-phosphonium] pamoate, [TOPP]_2_[PAM], is presented in Figure 2. The synthetic approach was divided into three stages following the principles of chlorination of alcohols 1), quaternization reaction of tertiary phosphines 2) and anion metathesis 3).

The synthesis of the precursor for the hydrophobic side chain, namely, 1-chloro-8-phenyloctane, was conducted by reaction of 8-phenyloctan-1-ol with thionyl chloride (Eistetter and Rapp, 1982). The structure of this intermediate was confirmed by ^1^H-NMR (Supplementary Figure S1), showing the five aromatic hydrogen atoms at 7.30–7.23 ppm (m, 2H, H_arom_) and 7.21–7.13 ppm (m, 3H, H_arom_) as well as the two hydrogen atoms at the halogenated carbon at 3.62 (t, *J* = 6.6 Hz, 2H, –CH2–Cl). No further purification steps were necessary for the quaternization reaction of 1-chloro-8-phenyloctane with trioctylphosphine yielding trioctyl-(8-phenyloctyl)-phosphonium chloride [TOPP]Cl, which was synthesized following a similar synthesis route reported by Cieniecka-Rosłonkiewicz et al. (2005). This reaction could be conducted solvent free and the retrieved excess 1-chloro-8-phenyloctane was reusable for further synthesis. For decolorization experiments of [TOPP]Cl, a simplified procedure described by Earle et al. (2007) was applied. These decolorization attempts, however, were only marginally successful and NMR and MS spectra showed no improved purity, so that this procedure was not pursued further. ^1^H-NMR spectra (Supplementary Figure S2) confirmed the presence of five aromatic hydrogen atoms at 7.30–7.23 ppm (m, 2H, H_arom_) and 7.17 ppm (d, *J* = 7.5 Hz, 3H, H_arom_) as well as the absence of the two hydrogen atoms at the halogenated carbon at 3.62 (t, *J* = 6.6 Hz, 2H, –CH2–Cl) from the side chain precursor. The MS spectrum (Supplementary Figure S6) in the positive mode confirmed a peak at m/z of 559.54 corresponding to the cations structure (C_38_H_72_P^+^) and additionally shows a fragment at m/z = 426.44.

The final product, the ionic liquid di-[trioctyl-(8-phenyloctyl)-phosphonium] pamoate, [TOPP]_2_[PAM], was synthesized following the deprotonation metathesis route described for other phosphonium-based ionic liquids with chloride as anion (Kogelnig et al., 2008; Stojanovic et al., 2010; Platzer et al., 2017a; Pirkwieser et al., 2018), but were conducted in a microwave synthesizer to accelerate the reaction time. ^1^H-NMR spectra (Supplementary Figure S3) showed peaks at 7.30–7.23 (m, 4H, H_arom_) and 7.17 (d, J = 7.3 Hz, 6H) corresponding to the aromatic hydrogen atoms of the cation’s side chain. The other peaks at shifts at 8.21 (d, *J* = 8.6 Hz, 2H, H_arom_), 8.13 (s, 2H, H_arom_), 7.63–7.57 (m, 2H, H_arom_), 7.08 (ddd, *J* = 8.4, 6.7, 1.4 Hz, 2H, H_arom_) and 6.97 (dd, *J* = 7.3, 7.3 Hz, 2H, H_arom_) can be assigned to each two of the anion’s aromatic hydrogen atoms. The two hydrogen atoms at the carbon atom linking the two naphthoic ring systems was visible at 4.66 (s, 2H, C_arom_–CH_2_–C_arom_). Peaks at 1.64–1.17 ppm (m, 96H, –CH_2_–) and 1.02–0.67 ppm (m, 18H, –CH_3_) were assignable to a total of 114 hydrogen atoms of the cation’s octyl side chains, peaks at 2.56 ppm (t, *J* = 7.7 Hz, 4H, C_arom_–CH_2_–) and 2.16 ppm (td, *J* = 13.1, 7.5 Hz, 16H, P–CH_2_–) to the hydrogen atoms of the phenyloctyl side chain. The ^31^P NMR (Supplementary Figure S5) showed a peak at 33.8 ppm corresponding to the product; the peak at 46.02 ppm is an impurity of the reactant trioctylphosphine. The MS spectra of [TOPP]_2_[PAM] in the positive mode (Supplementary Figure S7) confirmed a peak at m/z = 559.54 corresponding to the cation’s structure (C_38_H_72_P^+^) as well as a fragment at m/z = 426.44. In the negative mode (Supplementary Figure S8), the main peak was found at m/z = 193.04, corresponding to the anion pamoate (C_23_H_14_O_6_^2-^). Additionally, adducts in form of the singly deprotonated anion (C_23_H_15_O_6_^−^) and fragments in the form of [C_38_H_72_P]^+^[C_23_H_14_O_6_]^2-^ at m/z = 945.62 were determined.

Elemental Analysis of the IL [TOPP]_2_[PAM] disclosed the following elemental composition: 77.21% carbon, 10.74% hydrogen, 6.47% oxygen and 4.33% phosphorus. The chloride content was calculated to be 0.30%, nitrogen and sulfur were below 0.05% and 0.02% respectively. This equals a difference to theory of 1.73% (C), 0.17% (H), 0.10% (O) and 0.22% (P). Detailed data on the single results of elemental analysis are given in Supplementary Table S1. The relatively high difference in carbon content can be ascribed to the remaining water- and potassium chloride content. The water content could not be removed by further drying due to the highly viscous nature of the product, and the remaining chloride content was not decreased after additional fivefold extraction with a 3:1 dichloromethane/water mixture. Neither water nor potassium chloride can be regarded as limiting factors for the proposed metal extraction abilities and were therefore considered acceptable for the intended applications. Furthermore, the chloride content is comparable to literature values for ionic liquids synthesized from chlorinated precursors. Accordingly, Pirkwieser et al. (2018) reported a chloride content of 0.15 wt% for the IL trihexyltetradecylphosphonium 3-hydroxy-2-naphthoate and Stojanovic et al. (2010) reported comparable values from 0.11–0.90 wt% for Aliquat^®^ 336- and Cyphos IL 101^®^-based functionalized ionic liquids following a similar synthetic route for anion metathesis. Further characterization of the properties of the synthesized products included the determination of the density and viscosity. These physico-chemical parameters of the IL [TOPP]_2_[PAM] are presented in Table 1.

**TABLE 1:** Physico-chemical parameters of [TOPP]_2_[PAM].

Chemical formula	C_99_H_158_P_2_O_6_
molecular weight	1,506.30 g mol^−1^
density (20°C)	0.988 g cm^−3^
viscosity (20°C)	43,092 mPa s
Cl^−^ content	0.30%

The density of the IL (0.988 g cm^−3^ at 20°C) is only marginally below that of water (0.998 g cm^−3^) (Tanaka et al., 2001). Density values <1 g cm^−3^ are in good agreement with previously reported data from phosphonium-based ionic liquids (Stojanovic et al., 2010; Pirkwieser et al., 2018). Two ionic liquids based on Cyphos^®^ IL 101, trihexyltetradecylphosphonium 3-hydroxy-2-naphthoate and trihexyltetradecylphosphonium salicylate have densities of 0.97 g cm^−3^ (25°C) and 0.925 g cm^−3^ (20°C). Also, the phosphonium-based ionic liquid methyltrioctylphosphonium 3-hydroxy-2-naphthoate has a slightly lower density of 0.91 g cm^−3^ at 25°C.

**FIGURE 3:**
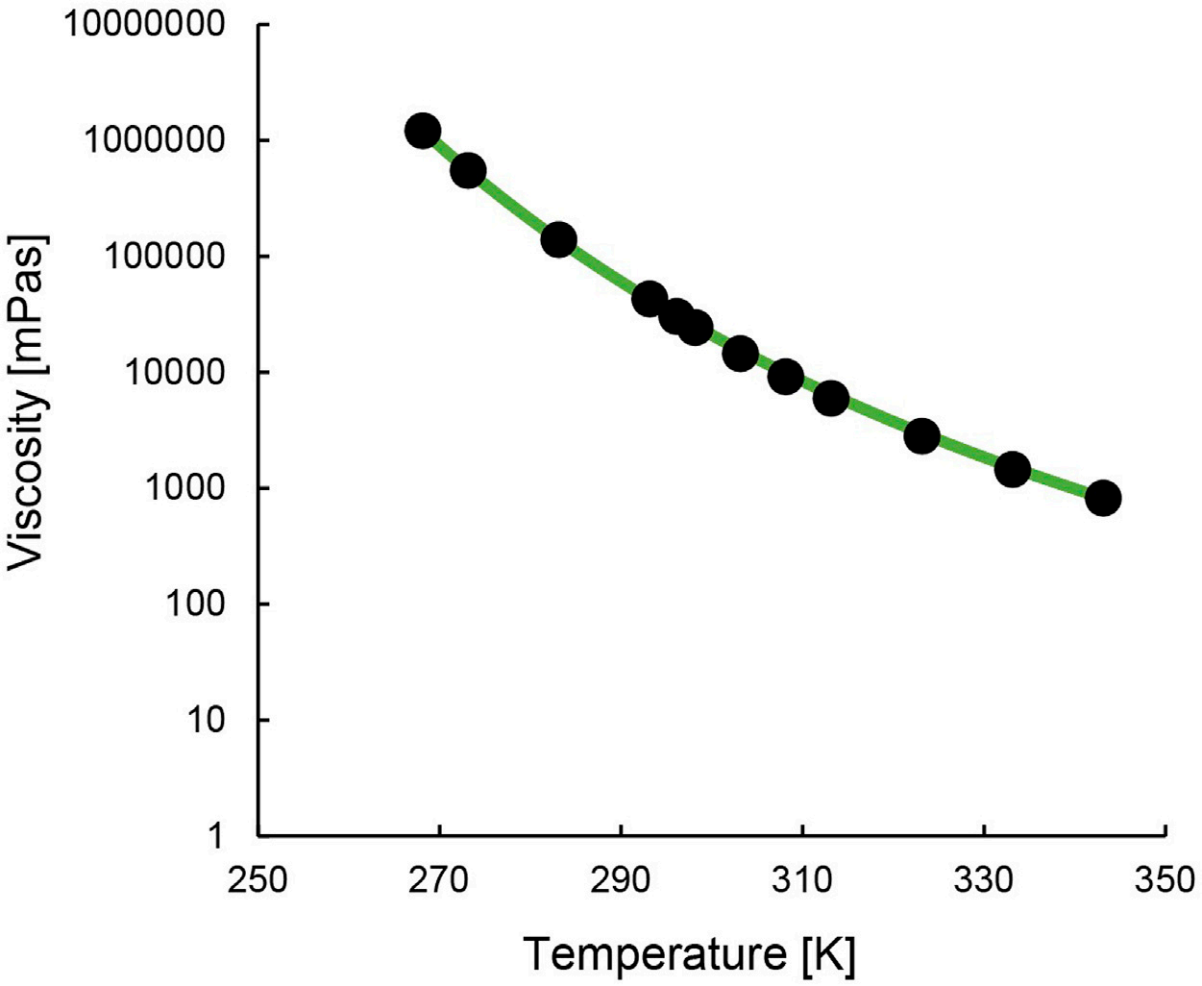
Steady state viscosity of [TOPP]_2_[PAM] in relation to temperature (black dots; green line = VFT fit).

Figure 3 and Supplementary Figure S9 show the IL’s change in viscosity with temperature. The temperature dependence can be described using the Vogel-Fulcher-Tammann (VFT) Equation 1 (Tao and Simon, 2015). The Vogel temperature, T_0_, is the temperature at which the viscosity of the material diverges to an infinite value. The results (Supplementary Table S2) show that this temperature is reached earlier for the precursor [TOPP]Cl than for [TOPP]_2_[PAM]. The constant A represents the asymptotic value of the viscosity (10^A^ mPa s), while B is a material-specific parameter. Their numerical values are comparable to those reported in earlier studies for ILs (Pogodina et al., 2011).

[TOPP]_2_[PAM] exhibited a temperature dependence ranging over three orders of magnitude from 1,000 to 1,000,000 mPa s. At 20°C, [TOPP]_2_[PAM] exhibited a viscosity of 43,092 mPa s, while at 50°C the value was lower (2,809 mPa s). [TOPP]_2_[PAM] was found to show a significantly higher viscosity than previously reported ILs such as [P_66614_][BTB] at 3,000 mPa s (25°C) or others (Leyma et al., 2016). However, [TOPP]_2_[PAM], despite showcasing very high viscosities at lower temperatures, exhibited fluid-dominated behaviour. This was confirmed by amplitude and frequency sweeps ([TOPP]_2_[PAM] in Supplementary Figure S10). Consistent with the viscosity measurements, both moduli dropped with increasing temperature, indicating an overall softer and less stiff material at higher temperatures. Both frequency and amplitude sweeps (Supplementary Figure S10), at both temperatures, were characterised by the viscous modulus being greater than the storage modulus. Thus, a viscous response of the material dominated the overall response to deformation.

### 3.2 Extraction of scandium, thorium and uranium

**FIGURE 4:**
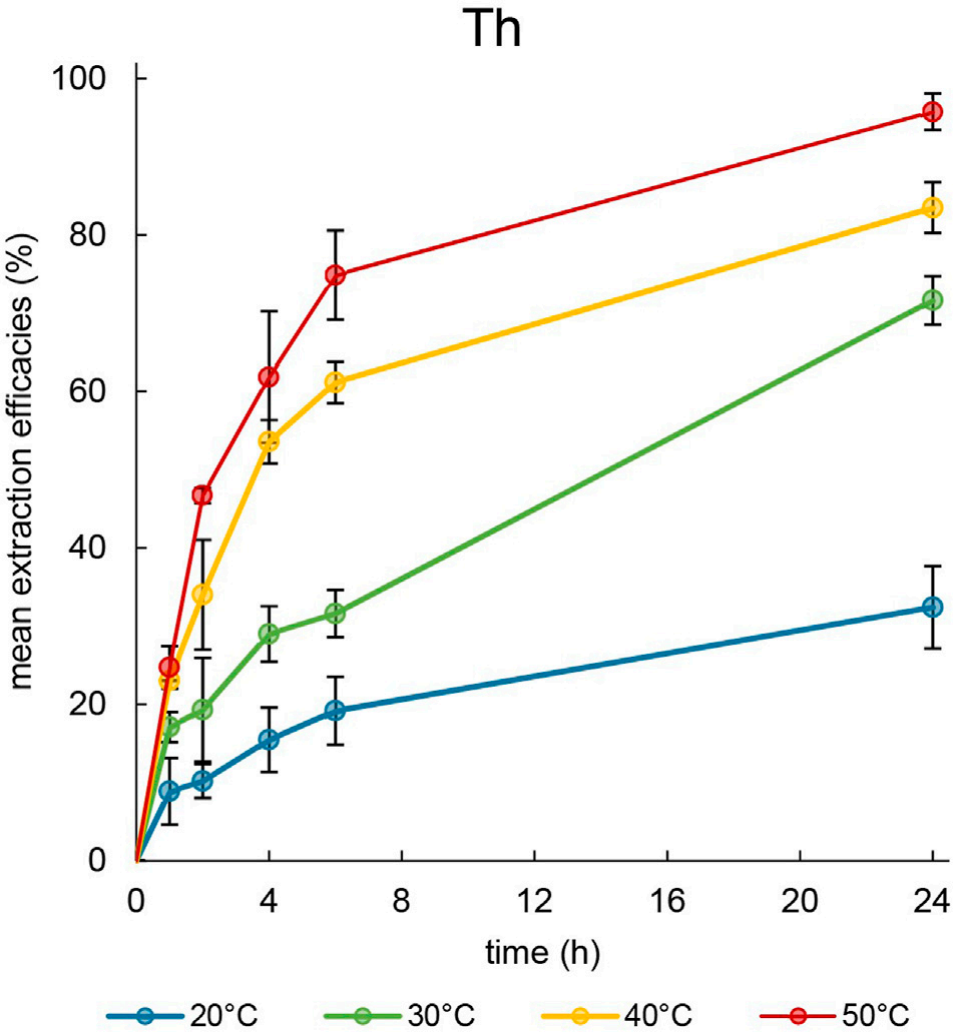
Mean extraction efficacies (±SD) over time using 100 mg [TOPP]_2_[PAM] to extract Th (10 mg L^−1^) from 20 mL aqueous phase (pH = 1.00) at different temperatures (n = 3).

The first extraction experiments were performed using the element Th to determine the influence of various temperatures. Nitric acid (2%) was used as the extraction matrix, and the pH was set to 1.00 ± 0.03 to ensure stability of the metal in the solution during the entire experiment. The metal concentration was measured after 1, 2, 4, 6 and 24 h. Even after 24 h, the two phases remained separated during the extraction process and no emulsification or third-phase formation was observed (Supplementary Figure S11). The corresponding extraction efficacies at 20, 30, 40°C and 50°C were calculated and are presented in Figure 4.

At 20°C, only 32.4% ± 5.3% of dissolved metal were extractable over a period of 24 h, whereas at higher temperatures 71.7% ± 3.1% (30°C) and 83.5% ± 3.2% (40°C) were extracted. At 50°C, 95.8% ± 2.3% of Th was removed from the aqueous phase, with about 75% already having been extracted after 6 h. The low extraction efficacy at 20°C can be ascribed to the high viscosity of the IL at this temperature. Reducing the viscosity by increasing the temperature yielded higher extraction efficacies and a faster time-dependent extraction. These observations agree with the findings of Mishra and Devi (2018). Those authors used Cyphos^®^ IL 104 to extract Eu^3+^ at various temperatures and explained the increased efficacies at higher temperatures with a decreased viscosity of the IL and an accelerated mass transfer. This positive correlation between temperature and extraction was also reported by Sert and Yusan (2023), emphasizing the decrease of the IL’s viscosity and the endothermic shift of the extraction equilibrium with increasing temperature. They extracted Th(IV) at 50°C from an acidic aqueous phase using the IL Cyphos^®^ IL 101. Their experimental setup differed from ours in the ratio of IL to aqueous phase: 0.5 g of IL and only 2 mL of aqueous phase. Nevertheless, they also adjusted the aqueous phase and its pH with diluted nitric acid. From a 50 mg L^−1^ Th solution, those authors were able to extract 95.5% ± 3.5% using Cyphos^®^ IL 101. Based on the volume and Th concentration they used (Sert and Yusan, 2023), [TOPP]_2_[PAM] is directly able to compete with Cyphos^®^ IL 101. In regard of the extraction mechanism, Sert and Yusan (2023) proposed a co-extraction of Th(IV) with nitrate, justified with the necessary electrical neutrality during the phase transition. Skanthakumar et al. (2017) showed that nitrate forms bidentate coordinated complexes with Th in the form of Th(NO_3_)_n+1_^(3−n)^, resulting in a dissolved complex in the form of a monovalent cation. They conclude that these findings are relevant for all tetravalent metal ions in nitrate solutions, regardless whether the metal is a transition metal, a lanthanide or actinide. These findings agree with the proposed co-extraction of a positively charged Th species together with nitrate as suggested by Sert and Yusan (2023). However, when using the Aliquat^®^ 336 derivative [A336][NO_3_] to extract of Th from a HNO_3_-containing aqueous solutions in different concentrations, Fu et al. (2021) described the formation of an IL trimer in the form [(A336)_2_(NO_3_)]^+^[A336(NO_3_)_2_]^-^, which is able to extract Th in the form of a pentanitrato complex [(Th)(NO_3_)_5_]^-^. In our work, we set the pH to 1.00 ± 0.03 to extract Th and remeasured the pH in the aqueous phase after the respective extraction times. This resulted in no change in the aqueous phase, and the pH remained constant at 1.00 ± 0.03. Considering that anion exchange as an extraction mechanism presumably alters the pH of the aqueous phase by releasing pamoate either during the formation of a [(TOPP)_2_(NO_3_)]^+^[TOPP(NO_3_)_2_]^−^ trimer or directly as result of the exchange with two equivalents of aqueous [(Th)(NO_3_)_5_]^−^, we do not presume anion exchange as the extraction mechanism in our experimental setup. Rather, we propose a neutral coextraction of thorium and nitrate. Nevertheless, the different findings described in the literature call for more extensive studies regarding the extraction mechanisms when extracting Th using ILs.

**FIGURE 5:**
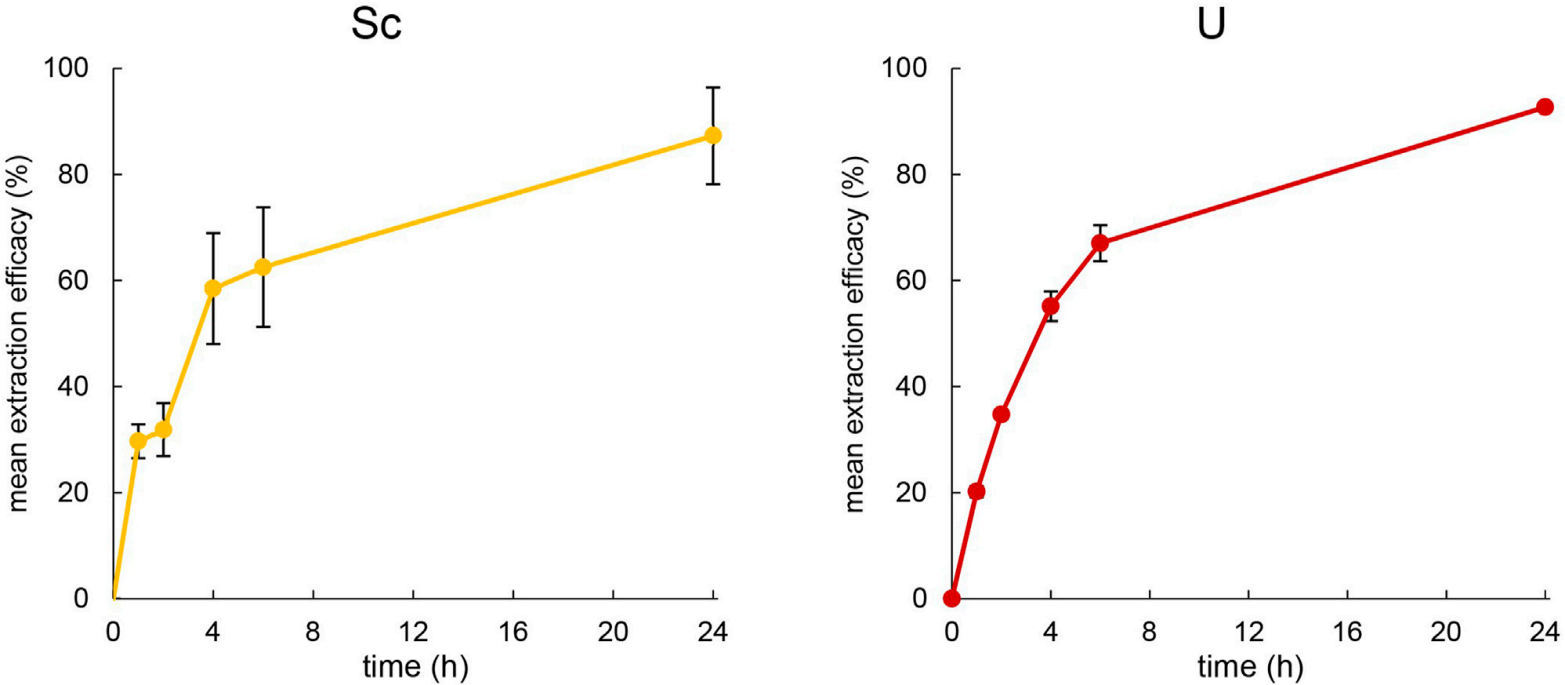
Left: Mean extraction efficacies (±SD) over time using 100 mg [TOPP]_2_[PAM] to extract Sc (10 mg L^−1^) from 20 mL aqueous phase (pH = 3.00) at T = 50°C (n = 3). Right: Mean extraction efficacies (±SD) over time using 100 mg [TOPP]_2_[PAM] to extract U (20 mg L^−1^) from 20 mL aqueous phase (pH = 1.00) at T = 50°C (n = 3).

As an extraction temperature of 50°C proved suitable to extract metals at concentrations of 10 mg L^−1^, the same experimental setup was used for Sc and U. For U, the extraction at 50°C was comparable to that of Th. However, at pH = 1.00 ± 0.03, Sc was not extracted from the identical, nitrate-containing aqueous phase. When increasing the pH to 3.00 ± 0.03, Sc could be extracted from the aqueous phase over 24 h. The time course of the extraction of U at pH = 1.00 ± 0.03 and Sc at pH = 3.00 ± 0.03 are presented in Figure 5.

Uranium was extracted very similarly to Th at 50°C, showing an extraction efficacy of about 75% after 6 h, peaking at 92.7% ± 0.3% after 24 h. During extraction, the pH remained stable and unchanged at pH = 1.0, implying a mechanism similar to that of Th. Srncik et al. (2009) used various Aliquat^®^ 336-based ILs featuring the three different, functionalized anions thiosalicylate, thiocyanate and methionate to extract U from aqueous uranyl nitrate solutions of 3.6 mg L^-1^. They successfully demonstrated the use of thiosalicylate and thiocyanate as functionalized anions of ILs, achieving extraction efficacies of 98% ± 1% after overnight extraction of U. Scheiflinger et al. (2019) used the functionalized anions anthranilate, 2-hydroxy-5-nitrobenzoate and 4-aminosalicylate combined with the cation of Cyphos^®^ IL 101, namely, trihexyltetradecylphosphonium, for U extraction. They extracted thereby between 92% and 99% U from 10 mL of an 11.6 mg L^−1^ aqueous extraction matrix at varying pH, highlighting that the extraction was already successfully completed after 2 h. Compared to the literature, the high suitability of using ILs with functionalized anions for U extraction was also confirmed using [TOPP]_2_[PAM] (Srncik et al., 2009; Scheiflinger et al., 2019). Compared to the fast extraction reported by Scheiflinger et al. (2019), however, the performance of [TOPP]_2_[PAM] was attenuated. We ascribe this, as in the case of Th, to the high viscosity of [TOPP]_2_[PAM], which decelerates the time-dependent mass transfer (Mishra and Devi, 2018; Sert and Yusan, 2023).

Sc extraction exhibited a comparable pattern to that of Th and U during the first 4 h, resulting in 58.5% ± 10.5% extraction efficacy. The increased efficacy then appeared less pronounced, peaking at 87.3% ± 9.1% at pH = 3 ± 0.03. Interestingly, the mean pH in the extraction matrix increased slightly to 3.33 ± 0.02 after 1 h, 3.42 ± 0.02 after 4 h and 3.50 ± 0.34 after 24 h during Sc extraction. The increase in pH in the extraction matrix and its correlation to extraction efficacy argue for explaining the extraction via an ion exchange extraction mechanism. The pK_a_ values of pamoic acid are pK_a1_ = 2.51 and pK_a2_ = 3.1 (Stahl and Wermuth, 2002), indicating that the pamoate anion is a weak base with the potential of altering the pH in the aqueous extraction matrix. Reports in the literature on the extraction mechanism are contradictory. Kaim-Sevalneva et al. (2024) reported the extraction of Sc via neutral extraction using the two quaternary-ammonium-based ILs [N333 MeOAc][Tf_2_N] and [N444 MeOAc][Tf_2_N]. Those authors described the formation of a Sc(III)-IL complex in the aqueous phase, which subsequently migrates into the organic phase, yielding in the neutral metal complex Sc [Tf_2_N]_3_. Using [Tf_2_N]^-^ as functional anion to extract Sc from aqueous solutions (pH = 3 and 0.5 mol L^−1^ NO_3_^−^) was also demonstrated by Su et al. (2024). Their findings imply cation exchange as the mechanism, forming with the IL lauryl betaine bis (trifluoromethanesulphonyl)imide, [Laur][Tf_2_N] a neutrally charged complex in the form of Sc([Laur][Tf_2_N]_3_)] and releasing three protons (H^+^) into the aqueous phase. Their observation of a significantly increased extraction efficacy when raising the pH from 2 to 3 is consistent with our findings and reflects the decreased competition between metal ion and protons in the aqueous solution. Note, however, their proposed release of H^+^ into the aqueous phase should lower the pH in the aqueous phase, a phenomenon that was not described by the authors and furthermore contradicts our findings. Based on the increase of pH during Sc extraction in our experimental setup, a more plausible extraction mechanism is that either the anion pamoate is released into the aqueous phase or H^+^ is extracted into the IL. Nevertheless, the reported extraction efficacies for Sc from comparable aqueous matrices using [N444 MeOAc][Tf_2_N] or [Laur][Tf_2_N] are–at 95.9% or 98.7%, respectively–slightly higher (Kaim-Sevalneva et al., 2024; Su et al., 2024), which potentially reflects the different metal concentrations and matrix-to-IL ratios used for extraction.

### 3.3 Selective extraction of scandium and thorium in the presence of rare earth elements

The extraction of REE, namely, Y, La, Ce, Eu, Ho and Lu from the 2% nitric acid extraction medium at pH = 1.00 or pH = 3.00 was not successful. The general assumption is that REE are extracted from nitrate-rich media in the form of penta- or hexanitrato complexes via anion exchange (Larsson and Binnemans, 2016; Onghena et al., 2018). We hypothesize that the success of such an anion exchange depends highly on the solubility of the anion in the aqueous phase. In our previous work, we successfully extracted La, Ce, Nd, Ho and Lu from the identical nitric acid media at pH = 2.5 using the IL [P_66614_][HNA] (Gradwohl et al., 2023). Thereby, pH changed in the extraction matrix after extraction due to the anion [HNA]^-^ (3-hydroxy-2-naphthoate). This confirmed the anion exchange extraction mechanism as being responsible for the successful extractions (Gradwohl et al., 2023). The anion pamoate, however, is less soluble in aqueous solutions, especially at low pH values where it is protonated to the practically insoluble pamoic acid (Stahl and Wermuth, 2002). This probably explains the hindered ion exchange mechanism and therefore unsuccessful extraction of REE under acidic conditions. At higher pH values, *e.g.*, pH = 6.00 or pH = 7.00, REE extraction was partly successful. Nevertheless, 60%–80% of initially dissolved metals precipitated during the experiment and where not susceptible for extraction into the IL. We therefore decided not to further pursue lanthanide extraction in the present study. However, the inability to extract REE at low pH values directed us to experiments for the selective extraction of Sc and Th and thereby separation from the stated REE.

#### 3.3.1 Extraction of Sc in the presence of other REE

In the case of Sc, we prepared double-element solutions in nitric acid (2%), set to pH = 3.00 ± 0.03, containing 10 mg L^−1^ Sc and 10 mg L^−1^ Y, La, Ce, Eu, Ho or Lu respectively. The extraction experiments were carried out over 24 h. The corresponding extraction efficacies are presented in Table 2.

**TABLE 2:** Extraction efficacies (mean ± SD) and separation factors (SF) of Sc in the presence of the REE Y, La, Ce, Eu, Ho and Lu. The highest possible extraction efficiency was calculated using the confidence interval (95%) when no certain decrease in metal concentration was detectable. These extraction efficiencies are given as “≤ %”.

	e.e. Sc (%)	e.e. REE (%)	SF
Sc and Y	62.6 ± 1.3	≤2.0%	0.75 ⋅ 10^2^
Sc and La	83.2 ± 9.5	10.9 ± 1.7	0.46 ⋅ 10^2^
Sc and Ce	86.4 ± 13.4	≤5.3%	1.16 ⋅ 10^2^
Sc and Eu	93.4 ± 5.0	5.0 ± 2.0	3.83 ⋅ 10^2^
Sc and Ho	92.4 ± 9.3	3.1 ± 2.1	4.36 ⋅ 10^2^
Sc and Lu	85.5 ± 3.3	≤1.5%	3.49 ⋅ 10^2^

**FIGURE 6:**
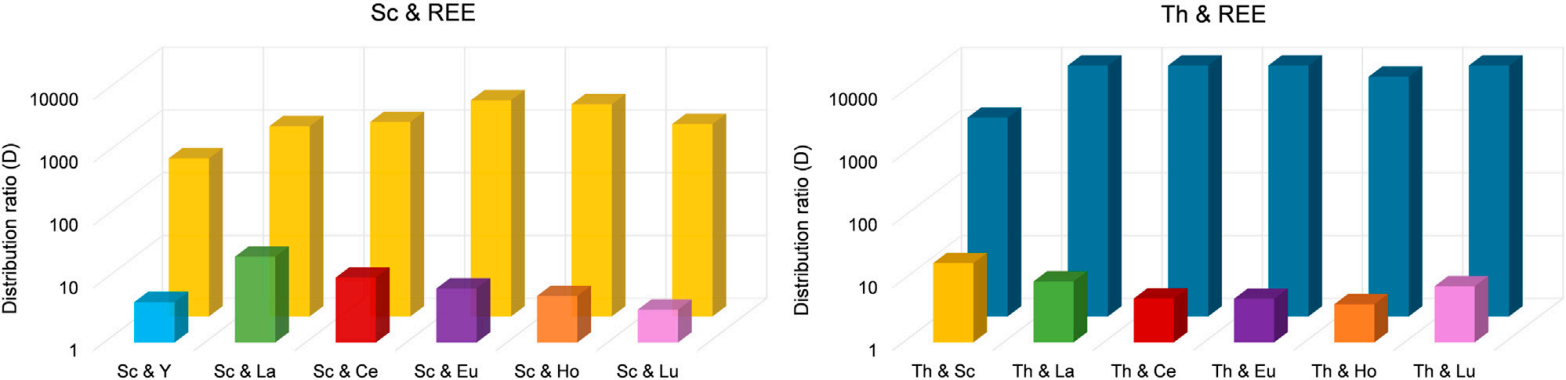
Distribution ratios for the separation of Sc from REE (left) and for Th from REE (right). To separate Sc from REE, we used an aqueous extraction matrix (20 mL) at pH = 3.00 containing 10 mg L^−1^ Sc and 10 mg L^−1^ Y, La, Ce, Eu, Ho or Lu (T = 50°C, 24 h, n = 3). For Th, we used an aqueous extraction matrix (20 mL) at pH = 1.00 containing 10 mg L^−1^ Th and 10 mg L^−1^ Sc, La, Ce, Eu, Ho or Lu (T = 50°C, 24 h, n = 3).

Interestingly, only 62.6% ± 1.3% of Sc was extracted in the presence of Y. Still, with an extraction efficacy ≤2.00% for Y, the separation via selective extraction can be considered highly effective. The lowest separation was achieved for Sc and La. When La was present, only 83.2% ± 9.5% of Sc was extracted and 10.9% ± 1.6% of La. Sc extraction was most successful in the presence of Eu and Ho (>90%), but also in the presence of Ce and Lu (>85%). The corresponding distribution ratios between the organic (IL) and aqueous phase for each element pair are presented in Figure 6 (left).

Even though the group of REE refers to Sc, Y and the lanthanides, the Sc atom is substantially smaller and forms much stronger complexes with ligands than the other REE. Moreover, hydrated Sc^3+^ contains six water molecules in its primary hydration sphere, whereas light and heavy REE have nine and eight hydration water molecules, respectively (Wood and Samson, 2006). These differences sufficiently explain selective extraction behavior. Su et al. (2024) found that the smaller ion radius of Sc compared to, *e.g.*, La or Lu and the resulting larger charge density enables the formation of stable complexes via interaction of Sc and the IL at pH values in between 2 and 3, thereby promoting selective extraction of Sc. This explanation is further supported by Kaim-Sevalneva et al. (2024). The low solubility of pamoate in the used matrix, which hinders extraction of lanthanide nitrato complexes via anion exchange, explains the selective extraction of Sc and demonstrates the exceptional separation behavior of [TOPP]_2_[PAM] for Sc from the other REE. Based on our observations, we propose the extraction mechanism to be ion exchange or the formation of Sc complexes with the anion pamoate (see chapter 3.4). The highest separation factors (SF, Table 2) were found for the REE Ce, Eu, Ho and Lu. These factors exceed the values found in the recent literature by up to two orders of magnitude (Kaim-Sevalneva et al., 2024), pointing to a clear advantage in using [TOPP]_2_[PAM].

#### 3.3.2 Extraction of Th in the presence of REE

The selectivity of [TOPP]_2_[PAM] towards Th was investigated using double-element solutions in 2% nitric acid at pH = 1.00 containing 10 mg L^−1^ Th and 10 mg L^−1^ Sc, La, Ce, Eu, Ho or Lu respectively. The corresponding extraction efficacies are presented in Table 3. Experiments on the selectivity of [TOPP]_2_[PAM] towards Th reveal an even higher separation potential. In all experiments using a mixture of Th and REE, Th was extracted with an efficacy >97%, with the exception of the double-element standard containing Sc and Th. In the latter, 88.2% ± 1.5% Th and 8.5% ± 1.5% Sc were extracted. Nonetheless, the results point to some separation of Th from Sc. As supported by the literature (Kaim-Sevalneva et al., 2024), pH values <2 are insufficient to extract Sc due to the competition of H^+^ and Sc(III) ions for uptake into the IL. Further reasons for the selectivity towards Th in contrast to REE can be associated with REE speciation in nitrate-containing aqueous phases. There, REE predominantly form negatively charged penta- or hexanitrato complexes (Larsson and Binnemans, 2016; Onghena et al., 2018), which are extractable via anion exchange exclusively under the used conditions. The low pH hinders the exchange of pamoate into the aquatic phase because the high proton concentration would immediately protonate it to the anti-soluble pamoic acid. In contrast, Th forms neutral complexes (see chapter 3.2), which are easily extracted. The corresponding distribution ratios of each element pair between organic (IL) and aqueous phase are presented in Figure 6 (right).

**TABLE 3:** Extraction efficacies (mean ± SD) and separation factors (SF) of Th in the presence of the REE Sc, La, Ce, Eu, Ho and Lu. The highest possible extraction efficiency was calculated using the confidence interval (95%) when no certain decrease in metal concentration was detectable. These extraction efficiencies are given as “≤ %”.

	e.e. Th (%)	e.e. REE (%)	SF
Th and Sc	88.2 ± 1.5	8.5 ± 1.5	0.80 ⋅ 10^2^
Th and La	98.3 ± 1.1	≤4.5%	1.21 ⋅ 10^3^
Th and Ce	99.4 ± 0.3	≤2.6%	6.68 ⋅ 10^3^
Th and Eu	99.2 ± 0.6	≤2.6%	4.76 ⋅ 10^3^
Th and Ho	97.1 ± 1.3	≤2.1%	1.63 ⋅ 10^3^
Th and Lu	98.3 ± 0.8	≤4.0%	1.42 ⋅ 10^3^

Highly effective separation was shown for Th and La using [TOPP]_2_[PAM]: 98.3% ± 1.0% of Th were extracted, while La was extracted to an extent of only ≤4.5%. Good separation of Th(IV) from La(III) and Ce(III) from nitrate-containing solutions using the IL Cyphos^®^ IL 101 was reported by Sert and Yusan (2023). Those authors achieved best results using 50 mg L^−1^ Th and either 50 mg L^−1^ La(III) or Ce(III), yielding 96% ± 1% Th and only 0.8% ± 0.2% Ce(III) and 8.9% ± 1.1% La(III). The separation of Th(IV) and Ce(IV) was not successful using Cyphos^®^ IL 101. When using [TOPP]_2_[PAM] in our work, Th was separated efficiently from Ce(IV), removing 99.4% ± 0.3% of Th and ≤2.6% of Ce. Our results point to a clear advantage of [TOPP]_2_[PAM] compared to Cyphos^®^ IL 101 in the applicability in separating Th from REE, both for the separation from La(III) and Ce(IV). The same grade of separation effectiveness as for Th and Ce was also achieved for Th and Lu. The highest separation factors (SF, Table 3) for Th from REE were determined for Ce(IV) and Eu (III), closely followed by La(III), Ho(III) and Lu(III), while the factor for Sc was two orders of magnitude lower. These separation factors are comparable to recent literature (Fu et al., 2021; Sert and Yusan, 2023).

### 3.4 Leaching of the ionic liquid [TOPP]_2_[PAM]

**FIGURE 7:**
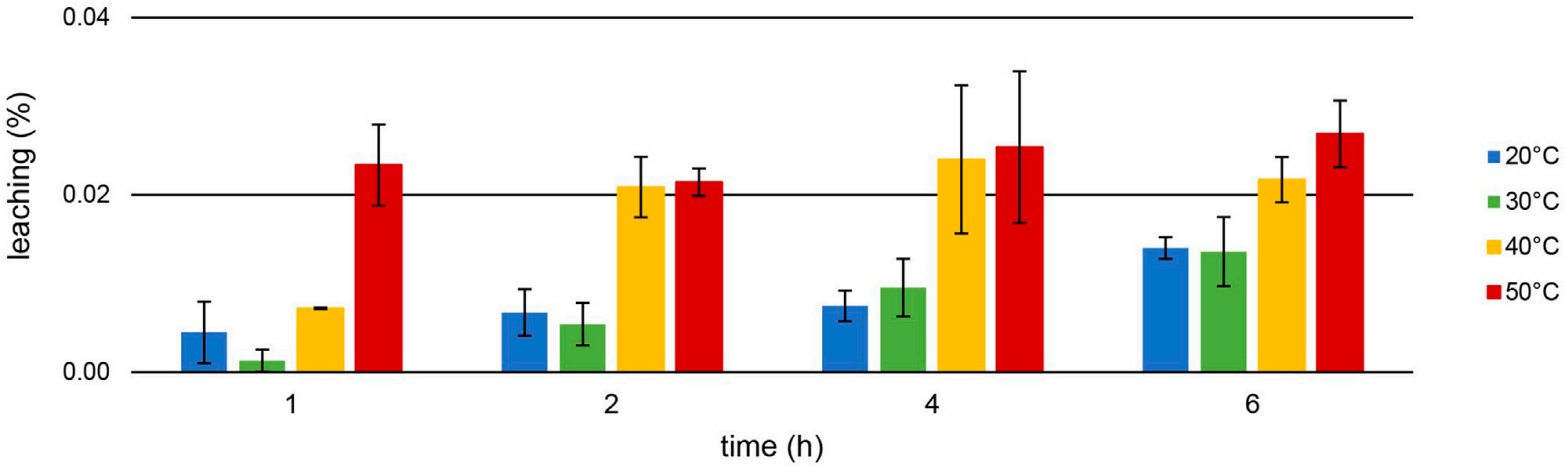
Mean leaching (%) ± SD of [TOPP]_2_[PAM] (100 mg) into the aqueous phase (20 mL, pH = 1.00) during Th extraction (10 mg L^−1^) over time and at different temperatures (n = 3).

[TOPP]_2_[PAM] was designed to exhibit a highly increased hydrophobicity compared with commercially available ILs. A reduced loss of substance into the aqueous phase during extraction should be achieved, enhancing the reusability of the IL. Figure 7 presents the leaching (%) during the Th extraction at different temperatures after 1, 2, 4 and 6 h.

Leaching of the IL during Th extraction is correlated with temperature: lowest values were recorded at 20°C, highest at 50°C. For example, while a leaching of only 0.014% ± 0.001% at 20°C occurred after 6 h, 0.027% ± 0.004% was determined at 50°C. At all temperatures, the leaching increased more or less significantly over time, peaking at 0.134% ± 0.011% after 24 h at 50°C. This value represents a maximum leaching of 6.61 ± 0.47 mg L^−1^. Remarkably, this is up to two orders of magnitude lower compared to the leaching of, *e.g.*, the IL trihexyltetradecylphosphonium 3-hydroxy-2-naphthoate, [P_66614_][HNA] (Pirkwieser et al., 2018). The leaching for [P_66614_][HNA] was reported between 0.23% after 1 h and 0.27% after 24 h in aqueous matrices with neutral to slightly basic pH values. We used this IL in our previous work to extract, *e.g.*, Nd from a nitric acid (2%) matrix at pH = 2.5. Thereby, [P_66614_][HNA] leached to an extent of 0.99% ± 0.03% after 6 h of extraction at room temperature (Gradwohl et al., 2023). Scheiflinger et al. (2019) investigated the leaching of 10 different ionic liquids after the extraction of radionuclides from diverse aqueous matrices. They reported a leaching >1% for 9 out of the 10 used ionic liquids, underlying that this might merely be suitable for technical applications. For example, the ionic liquid methyltrioctylphosphonium anthranilate, [P_1888_][Ant], extracted U at pH = 2.6 with an efficiency of about 99%, but a TOC value of 135 ± 22 mg L^−1^ was determined in the matrix after the extraction. This value is two orders of magnitude higher than the 2.13 ± 0.42 mg L^−1^ total organic carbon (TOC) after 24 h of U extraction or the 5.22 ± 0.47 mg L^−1^ after 24 h Th extraction using [TOPP]_2_[PAM]. Our experiments conducted for comparison using the commercially available ILs Aliquat^®^ 336 and Cyphos IL 101^®^ in ultrapure water showed high leaching values: 1,594 ± 45.80 mg L^−1^ and 139.0 ± 17.10 mg L^−1^ (n = 3), respectively, had leached into the aquatic phase at 50°C after 24 h regardless of any ongoing metal extractions. These results highlight the significantly decreased leaching behavior of [TOPP]_2_[PAM].

**FIGURE 8:**
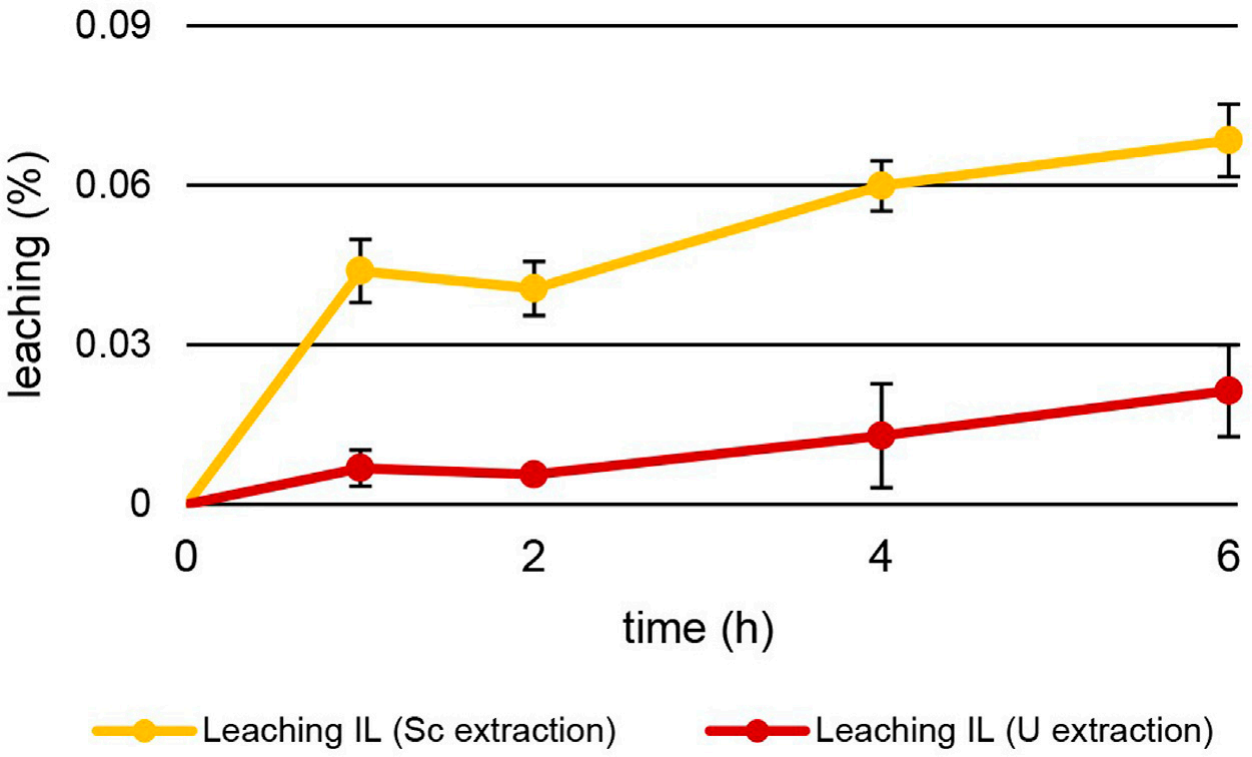
Mean leaching (%) ± SD of [TOPP]_2_[PAM] (100 mg) into the aqueous phase (20 mL) over time during the extraction of Sc (10 mg L^−1^, pH = 3.00) and U (20 mg L^-1^, pH = 1.00) at T = 50°C (n = 3).

Other than temperature, leaching also depended on the pH of the aqueous extraction matrix (Figure 8). The increase in leaching during U extraction mirrors the values during Th extraction. After 6 h at pH = 1.00 ± 0.03, 0.021% ± 0.009% of [TOPP]_2_[PAM] had leached into the aqueous phase at 50°C. During Sc extraction at pH = 3.00°C and 50°C, the behaviour was different. Already after 1 h of extraction, 0.044% ± 0.006% of IL had leached, twice as much as after 6 h of Th and U extraction. After 6 h of Sc extraction, 0.068% ± 0.007% of [TOPP]_2_[PAM] had dissolved into the aqueous phase. We hypothesize that this higher leaching has two explanations: 1) the higher pH of 3 in the extraction matrix might promote the solubility of the ionic liquid in general and especially of the anion pamoate; 2) the ongoing extraction mechanism might boost leaching. As discussed above, different extraction mechanisms (Fu et al., 2021; Sert and Yusan, 2023; Kaim-Sevalneva et al., 2024; Su et al., 2024) can be described for the same metal from similar aqueous compositions depending on the pH, the ILs and the competing ions in solution. In general, the metal extraction of ILs relies on the mechanisms of neutral extraction or ion exchange. One of these mechanisms can dominate or a balance between the two can occur (Janssen et al., 2015). Based on our results, we propose two different mechanisms for extraction, while conclusions regarding extraction equilibria or the extracted species are difficult. Th and U extraction appear to follow the principles of neutral extraction, whereas Sc is extracted via ion exchange or the complexation of the metal within the IL. The increased pH after the extraction of Sc indicates either an uptake of H^+^ into the IL or a neutralization of H^+^ in the aqueous phase. The H^+^ uptake would compensate a charge imbalance caused by the IL’s cation leaching into the aqueous phase. The neutralization of H^+^ in the aqueous phase, in contrast, would be caused by the protonation of the leached pamoate. The higher leaching during Sc extraction further points to ion exchange as the extraction mechanism, whereas the lower leaching rates during Th and U extraction support the concept of neutral extraction.

### 3.5 Loading capacity and back-extraction

**FIGURE 9:**
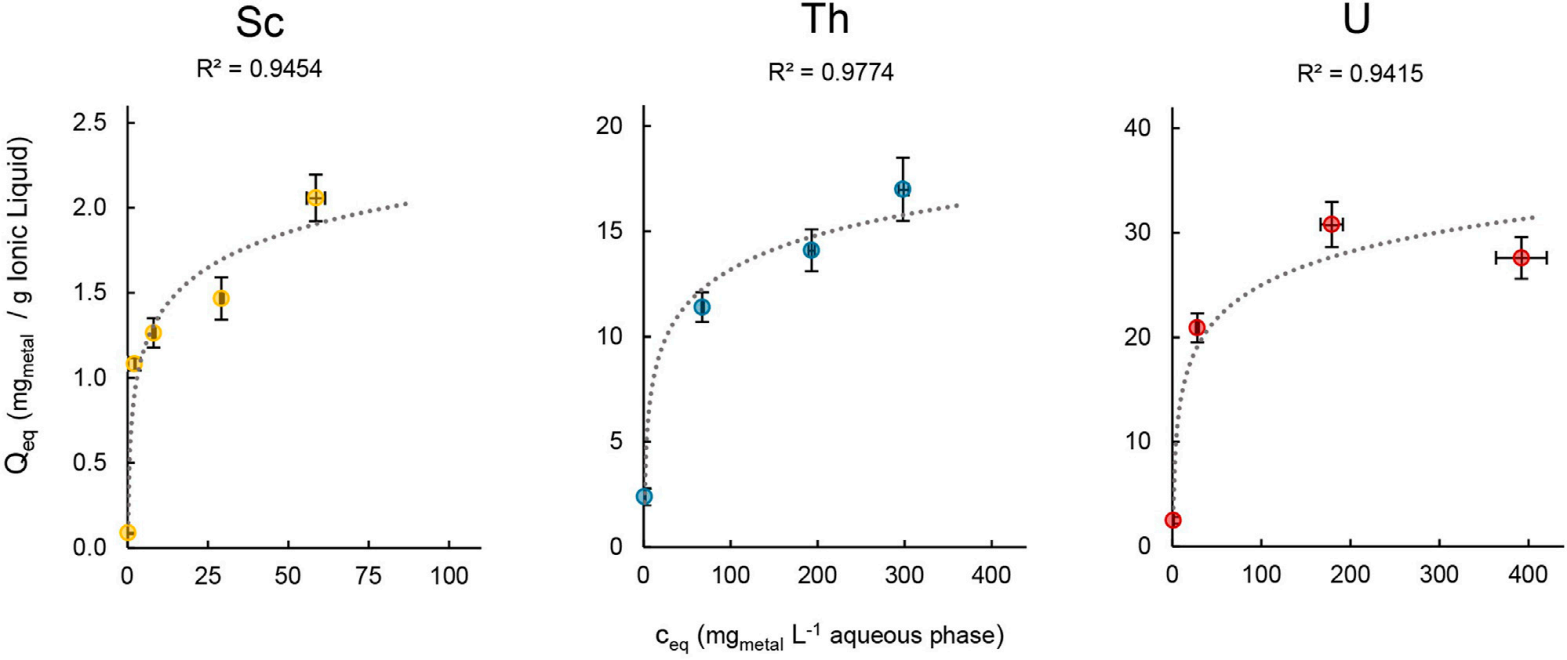
Mean metal uptake ±SD into [TOPP]_2_[PAM] (100 mg) of Sc, Th and U after 24 h in relation to the respective metal concentration in the aqueous phase (20mL, T = 50°C, n = 3).

The loading capacity for Sc, Th and U was determined in order to assess the maximal amount of metal that can be extracted into the IL. We therefore followed Mishra and Devi (2018) and Scheiflinger et al. (2019), and used aqueous extraction matrices with increasing metal concentrations ranging from 1–400 mg L^−1^ for the extraction experiments (Figure 9).

The maximal metal uptake per gram IL was 2.06 ± 0.20 mg for Sc, 17.0 ± 1.50 mg for Th and 30.8 ± 2.16 mg for U. In contrast, Scheiflinger et al. (2019) reported values of up to 200 mg U per g IL when extracting from a uranyl nitrate solution using the quaternary phosphonium-based IL [P_66614_][HNBA]. We assume that the differences between Sc (III), Th(IV) and U(VI) can be attributed to the speciation of the metals in solution, to their charge and to the extraction matrix composition. This agrees with further findings by Scheiflinger et al. (2019), who reported a higher loading capacity for the IL [P_66614_][HNBA]: about 300 mg U per g IL compared to 200 mg when extracting uranyl acetate instead of uranyl nitrate. Furthermore, a direct comparison of the loading capacity of ILs can be biased because various authors used different metal concentrations in the aqueous phase, different methods of evaluation, different ILs (diluted or undiluted) and different IL-to-aqueous-phase ratios. For example, Dong et al. (2016) used the IL [P_66614_][SOPAA] (747.23 g mol^−1^, 0.03 mol L^−1^) diluted in sulfonated kerosene (5 mL) to extract yttrium and reported a loading capacity of 0.16 g L^−1^. Recalculated to mg_metal_ per g IL, that value would be 7.14 mg Y per g [P_66614_][SOPAA]. Even though in dilution, this value is well comparable to our findings for Sc and Th. In our experimental setup, we can report the highest metal uptake for U, which is lower by a factor of 2 for Th and a factor of 15 for Sc.

Back-extraction and the recovery of metal into the aqueous phase was only partly successful. For this work, we used different nitric acid concentrations, following Platzer et al. (2017a), different concentrations of sulfuric acid or 0.03 M EDTA +0.5 M NaCl, following Sert and Yusan (2023), to obtain a first impression of the recoverability of Sc, Th and U after extraction. In the literature, both U and Th were back-extracted from an ionic liquid with anthranilate anion, [N_1888_][Ant], using 0.5 M HNO_3_ (Scheiflinger et al., 2019), while Th could also be recovered after extraction into [P_66614_]Cl at pH = 2.00 by EDTA in combination with NaCl as salting-in agent (Sert and Yusan, 2023). Our best experimental results were achieved for the back-extraction of Sc using nitric acid (10%): 82.7% ± 2.8%. This agrees well with the literature, *e.g.*, Kaim-Sevalneva et al. (2024), who reported 86% stripping efficiency when using 4 M H_2_SO_4_ for the quaternary ammonium-based ILs [N333 MeOAc][Tf_2_N] and [N444 MeOAc][Tf_2_N]. IL leaching into the back-extraction matrix amounted to 3.24% ± 0.02% and probably reflected the high acid concentrations. This argues for recovering Sc in several cycles using milder conditions with decreased efficiency but also decreased leaching. Th and U were less efficiently recovered. Sert and Yusan (2023) suggested using an aqueous solution containing 0.03 M EDTA +0.5 M NaCl for the back-extraction of Th. The authors achieved full recovery of the extracted Th under these conditions, with gradually less efficacy with increasing sodium chloride content. This back-extraction agent failed in our recovery experiments for Th and U; when using it for Sc, however, 40.7% ± 0.3% was back-extracted. Using sulfuric acid (5%) enabled only about 20% of Th to be back-extracted into an aqueous matrix. The values were even lower with the other back-extraction agents. For U, only about 10% could be recovered. The reason for these low efficiencies compared to other works remains unknown and will be the subject of future research.

## 4 Conclusion

The novel TSIL di-[trioctyl-(8-phenyloctyl)-phosphonium] pamoate, [TOPP]_2_[PAM], was synthesized at high yield and proved to be suitable for extracting Sc, Th and U from aqueous media. Best results (87.3% ± 9.1%) in terms of the extraction conditions for Sc were achieved at pH = 3.00, and at pH = 1.00 for Th (95.8% ± 2.3%) and U (92.7% ± 0.3%). Furthermore, we demonstrated a high selectivity for Sc in the presence of Y, La, Ce, Nd, Eu, Ho and Lu (separation factors up to 4.36 ⋅ 10^2^) and an even higher selectivity for Th in the presence of Sc, La, Ce, Nd, Eu, Ho and Lu (separation factors up to 6.68 ⋅ 10^3^). The high separation performance calls for further studies on the extraction mechanisms in order to validate initial experimental insights and promote our understanding of this novel compound. During all experiments, IL leaching was much lower compared to similar ILs, peaking at 0.134% ± 0.011% after 24 h during the extraction of Th at 50°C, albeit remaining far below that value during the first 6 h. Based on these promisingly low leaching values, we urge research on the extraction behavior of [TOPP]_2_[PAM] towards other metals, extraction experiments under environmentally relevant conditions, as well as studies on the (eco)toxicological behavior of this novel compound to clarify the IL’s potential usage as a greener solvent and extractant.

## Data Availability

The original contributions presented in the study are included in the article/Supplementary Material, further inquiries can be directed to the corresponding author.
