# Supplementary material for: Di-[trioctyl-(8-phenyloctyl)-phosphonium] pamoate: synthesis and characterization of a novel, highly hydrophobic ionic liquid for the extraction of scandium, thorium and uranium
Source: Front Chem. 2024 Nov 19;12:1502232. doi: 10.3389/fchem.2024.1502232 (PMC11611576; doi:10.3389/fchem.2024.1502232)
Supplement: Supplementary file 1 [file DataSheet1.docx]

Supplementary Material

# Supplementary tables

**Supplementary Table 1**. Results of elemental analysis of six different samples for C, H, N, S and O, of eight different samples for P and of 4 different samples for Cl. Average (%), standard deviation, theoretical content (%) and difference to the theoretical (%) content are given below the single results.

|  | **C (%)** | **H (%)** | **N (%)** | **S (%)** | **O (%)** | **P (%)** | **Cl (%)** |
| --- | --- | --- | --- | --- | --- | --- | --- |
|  | 77.54 | 10.81 | < 0.05 | < 0.02 | 6.41 | 4.32 | 0.425 |
|  | 77.39 | 10.80 | < 0.05 | < 0.02 | 6.18 | 4.30 | 0.233 |
|  | 77.51 | 10.83 | < 0.05 | < 0.02 | 6.47 | 4.19 | 0.280 |
|  | 77.33 | 10.73 | < 0.05 | < 0.02 | 6.63 | 4.36 | 0.256 |
|  | 77.30 | 10.71 | < 0.05 | < 0.02 | 6.47 | 4.59 |  |
|  | 76.18 | 10.56 | < 0.05 | < 0.02 | 6.68 | 4.30 |  |
|  |  |  |  |  |  | 4.28 |  |
|  |  |  |  |  |  | 4.27 |  |
| **Average** | 77.21 | 10.74 | < 0.05 | < 0.02 | 6.47 | 4.33 | 0.30 |
| **STDV** | 0.47 | 0.09 |  |  | 0.16 | 0.10 | 0.07 |
| **Theory** | 78.94 | 10.57 | - | - | 6.37 | 4.11 | - |
| **Difference** | 1.73 | 0.17 | - | - | 0.10 | 0.22 | 0.30 |

**Supplementary Table 2.**VFT fit parameters.

|  | **[TOPP]Cl** | **[TOPP]_2_[PAM]** |
| --- | --- | --- |
|  | *VFT Fit:* | *VFT Fit:* |
| **A** | -0.4006 | -1.63032 |
| **B** | 357.93004 | 829,53471 |
| **T_0_** | 182.31194 | 160.41042 |

# Supplementary figures

## ^1^H NMR spectra


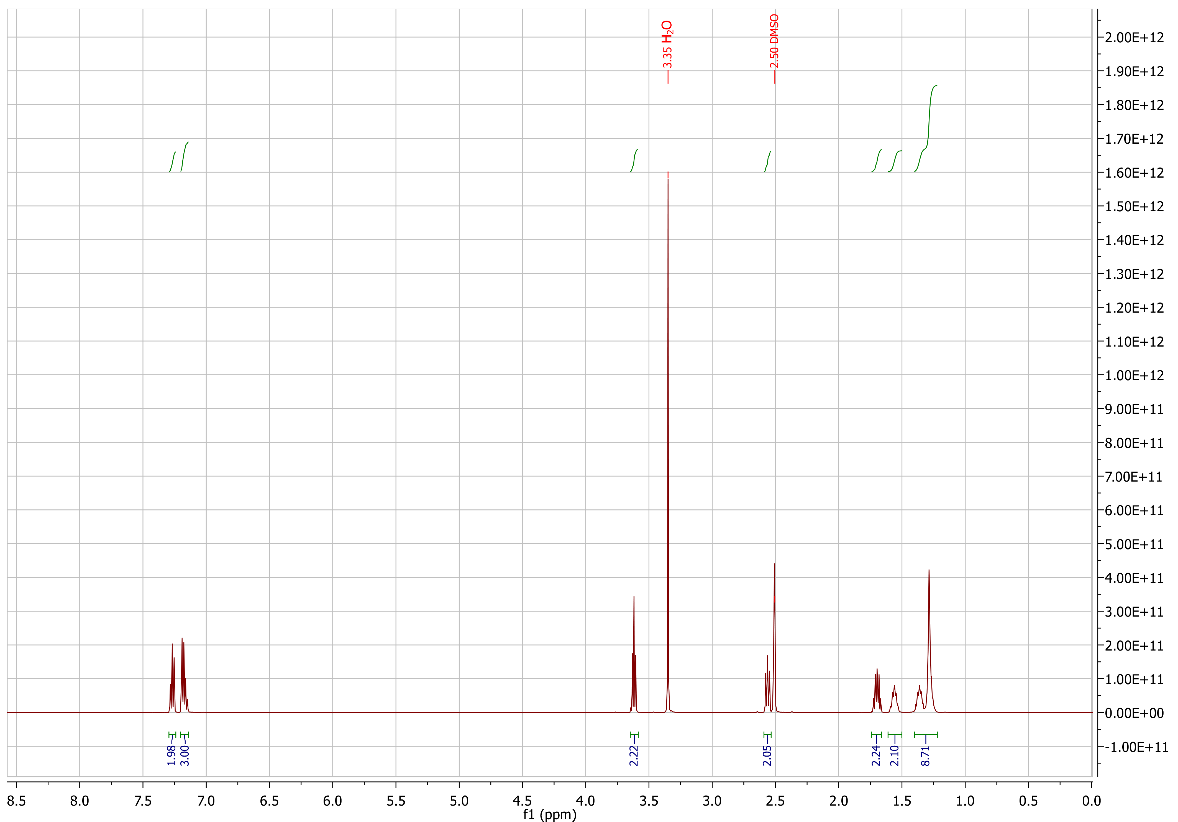


**Supplementary Figure 1.** ^1^H-NMR of 1-chloro-8-phenyloctane.


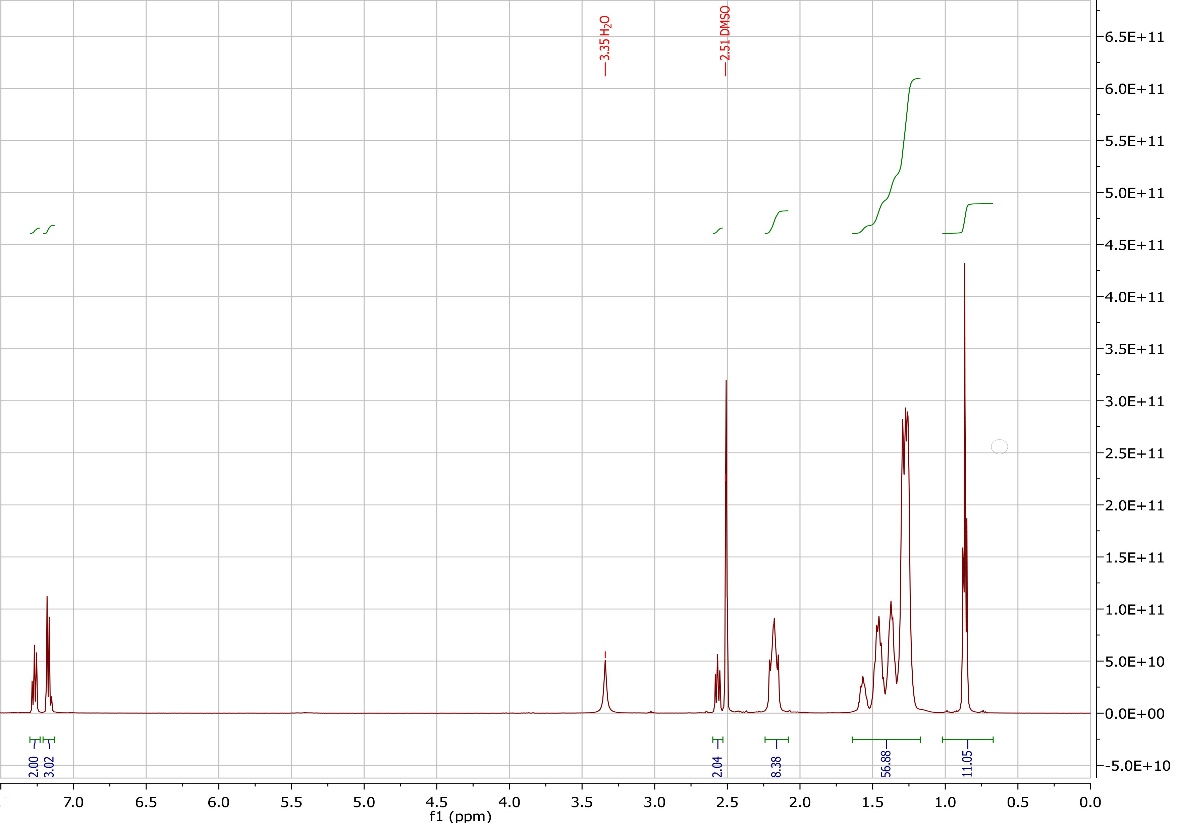


**Supplementary Figure 2.** ^1^H-NMR of trioctyl-(8-phenyloctyl)-phosphonium chloride, [TOPP]Cl.


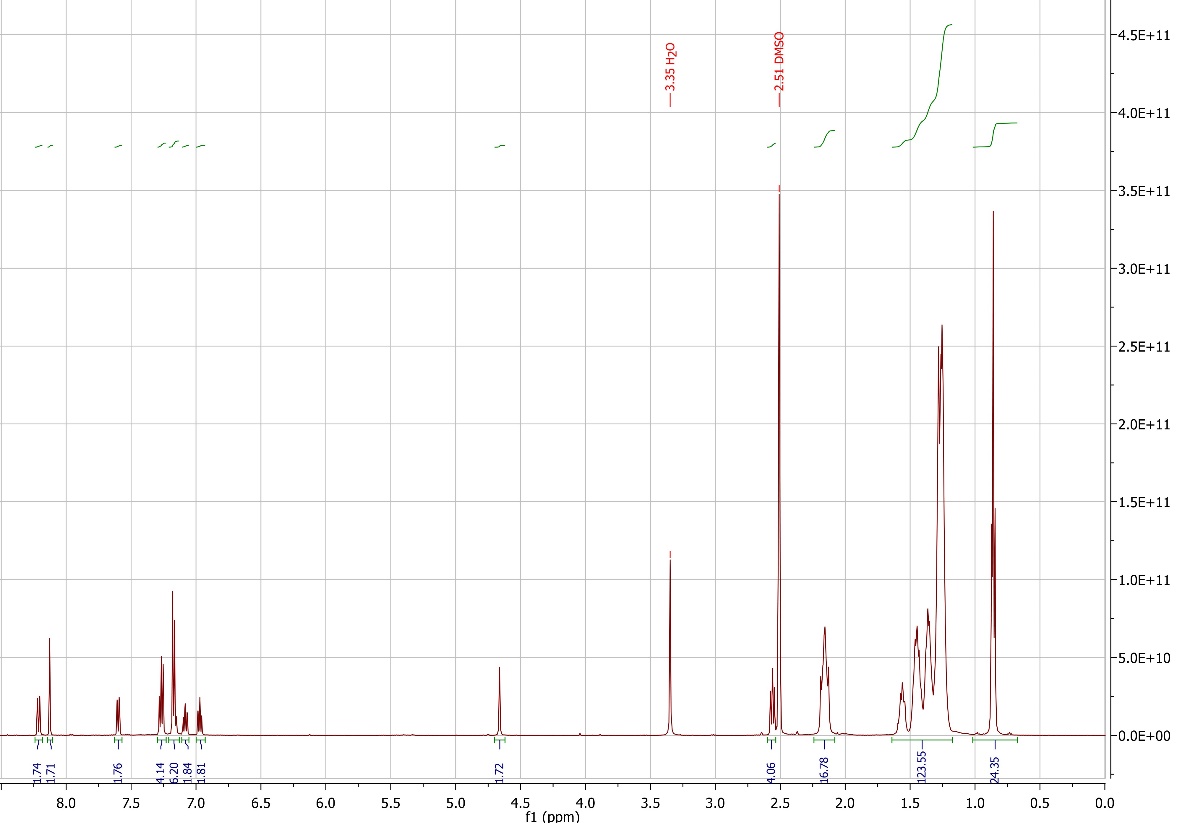


**Supplementary Figure 3.** ^1^H-NMR of di-[trioctyl-(8-phenyloctyl)-phosphonium] pamoate, [TOPP]_2_[PAM].

## ^31^P NMR spectra


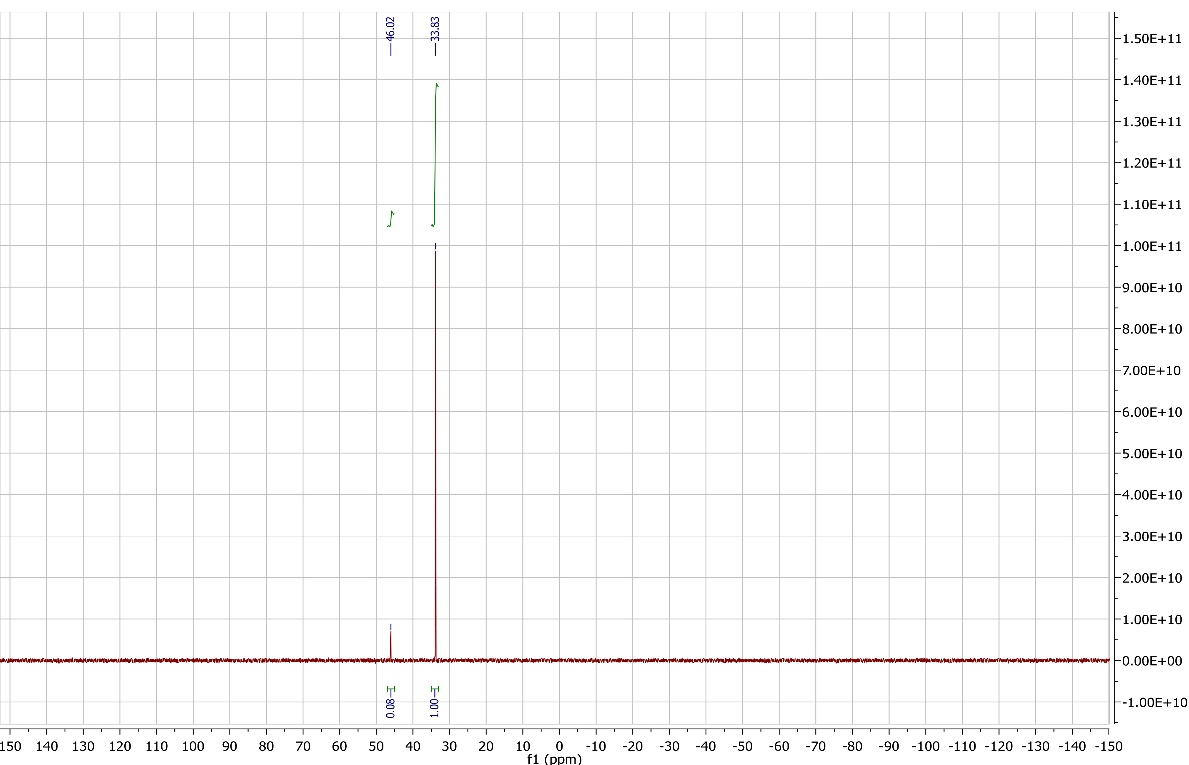


**Supplementary Figure 4.** ^31^P-NMR of trioctyl-(8-phenyloctyl)-phosphonium chloride, [TOPP]Cl.


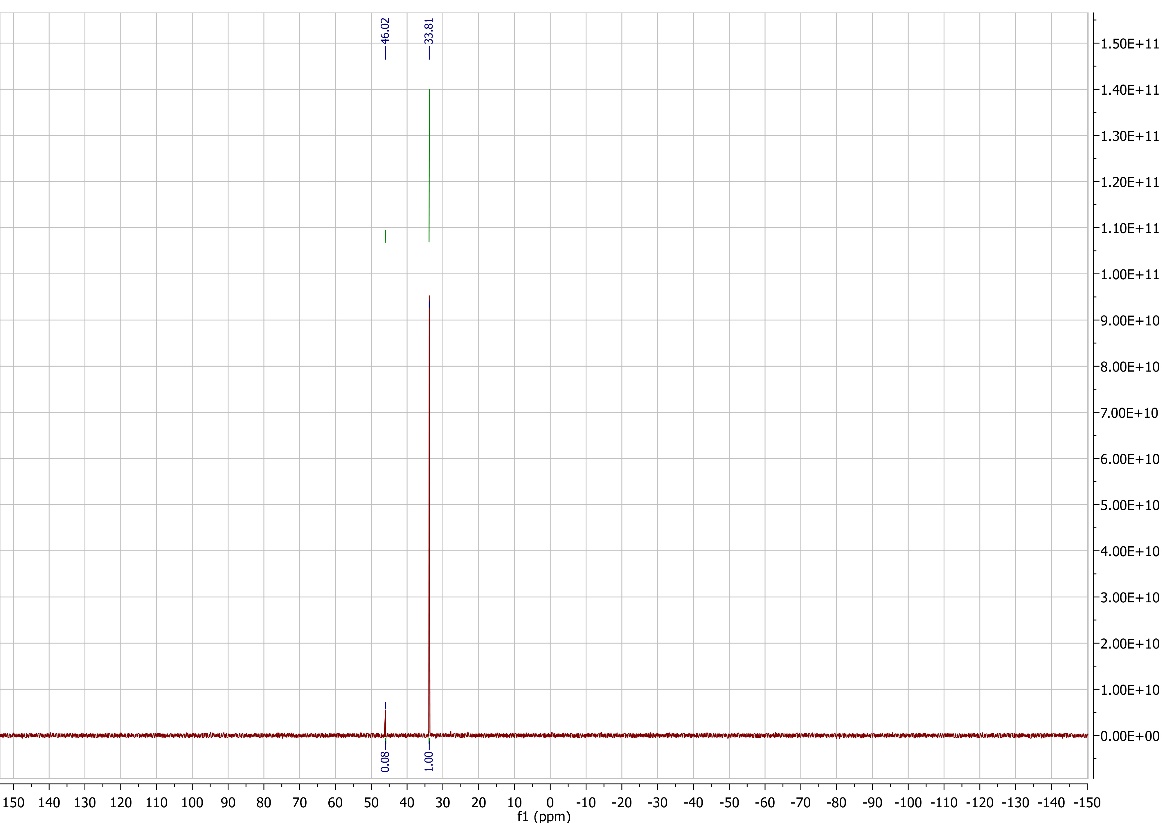


**Supplementary Figure 5.** ^31^P-NMR of di-[trioctyl-(8-phenyloctyl)-phosphonium] pamoate, [TOPP]2[PAM].

## MS spectra


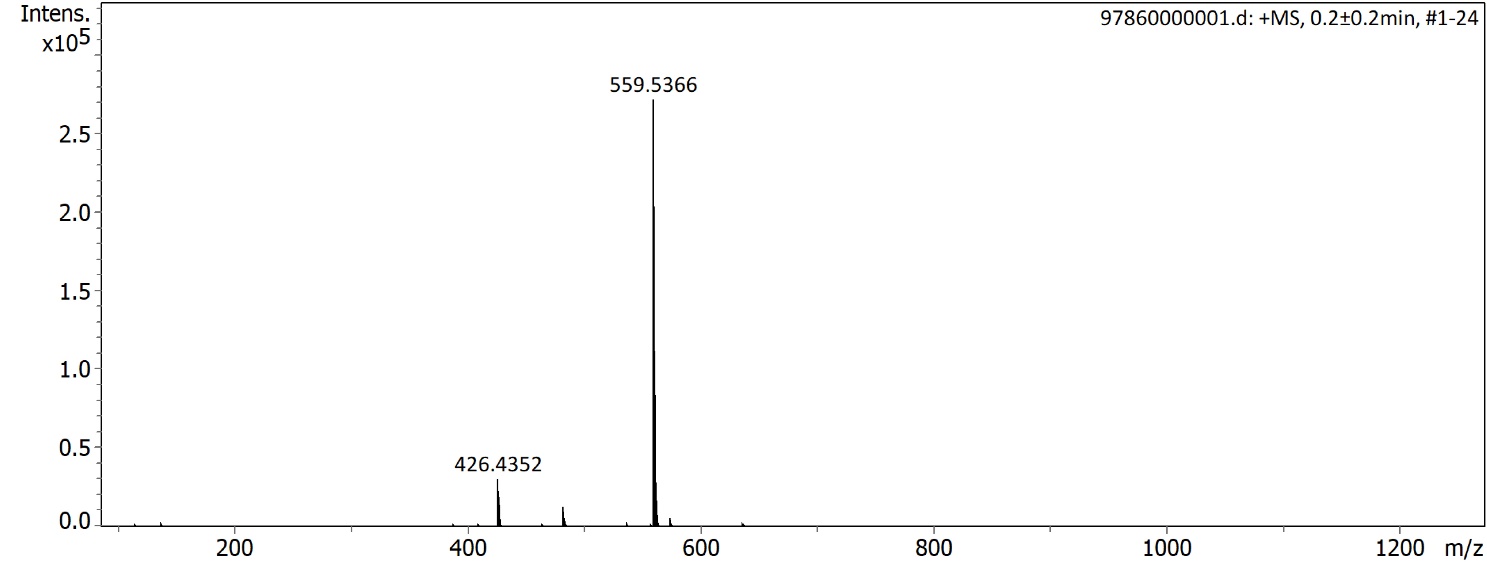


**Supplementary Figure 6.** +MS spectrum of trioctyl-(8-phenyloctyl)-phosphonium chloride, [TOPP]Cl.


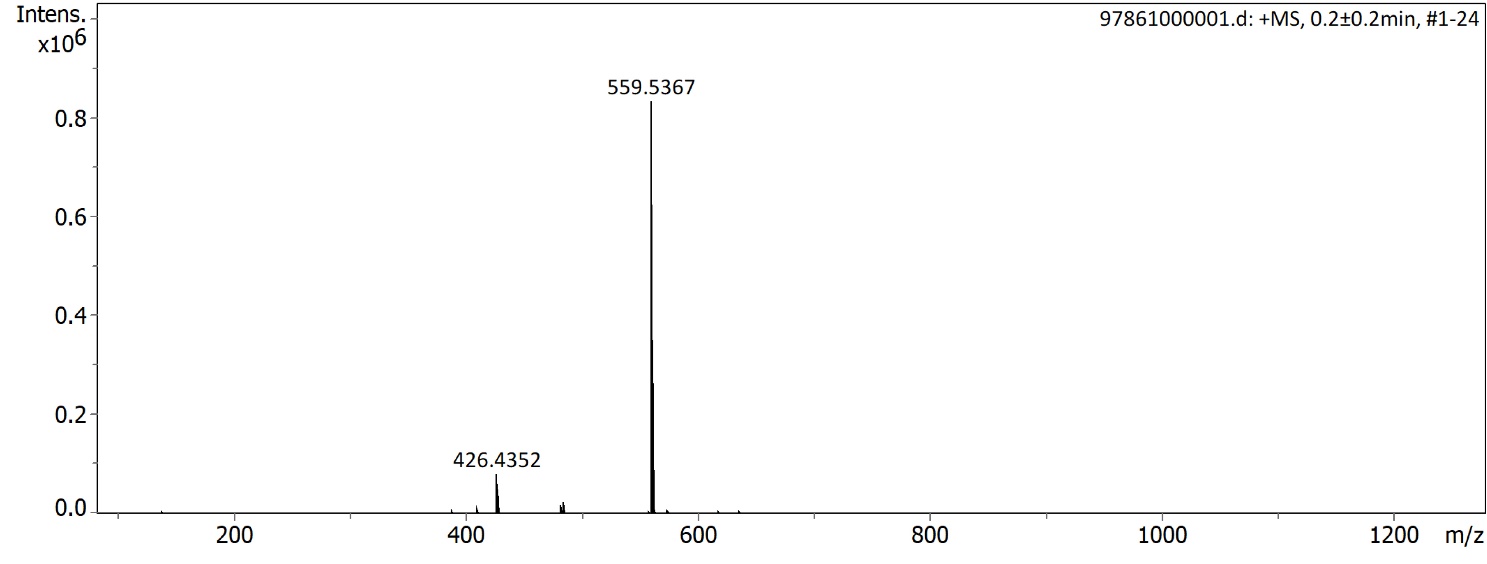


**Supplementary Figure 7.** +MS spectrum of di-[trioctyl-(8-phenyloctyl)-phosphonium] pamoate, [TOPP]_2_[PAM].


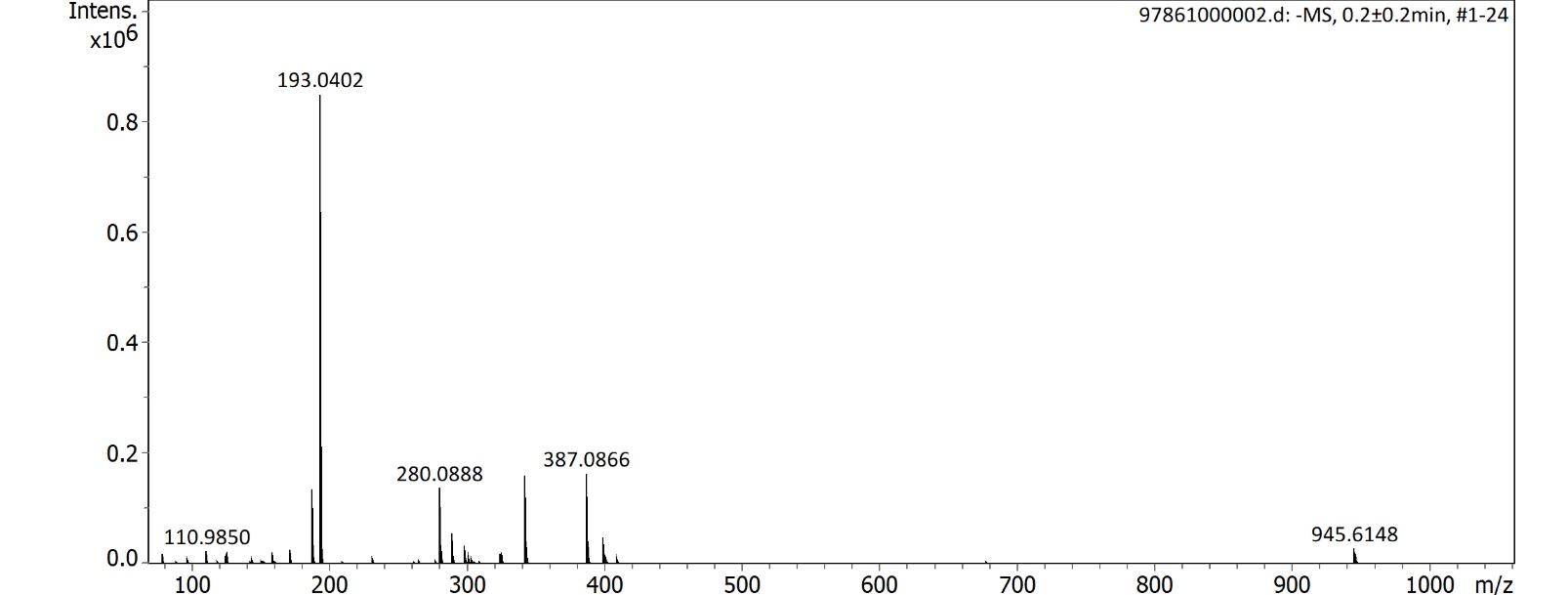


**Supplementary Figure 8.** -MS spectrum of di-[trioctyl-(8-phenyloctyl)-phosphonium] pamoate, [TOPP]_2_[PAM].

## Viscosity

**Supplementary Figure 9.** Steady state viscosity of [TOPP]Cl (grey dots; blue line = VFT fit).

**Supplementary Figure 10.** Left: Frequency sweep of [TOPP]_2_[PAM] at 20°C (blue) and 70°C (grey). Viscous modulus is shown as open symbols, elastic modulus as filled symbols. Right: Amplitude sweep of [TOPP]_2_[PAM] at 20°C (blue) and 70°C (grey). Viscous modulus is shown as open symbols, elastic modulus as filled symbols.

## Experimental


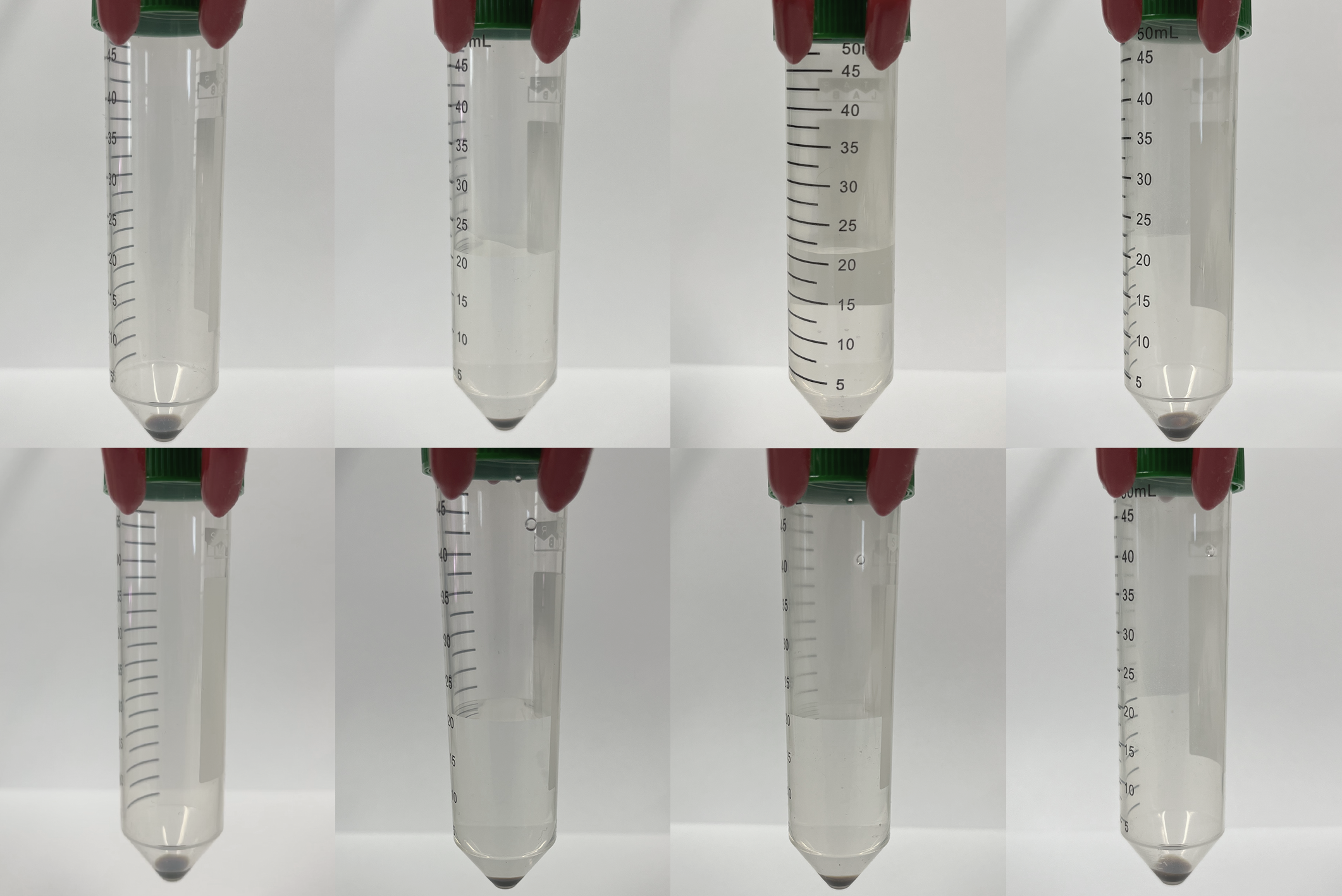


**Supplementary Figure 11.** Documentation of the extraction experiments at T = 50°C. Left: start of the extraction, right: after 24 hours. The two phases remained separated during the extraction process and no emulsification or third-phase formation was observed.
